# Variation in Pen-Level Prevalence of BRD Bacterial Pathogens and Antimicrobial Resistance Following Feedlot Arrival in Beef Calves

**DOI:** 10.3390/antibiotics13040322

**Published:** 2024-04-02

**Authors:** Jennifer N. Abi Younes, John R. Campbell, Simon J. G. Otto, Sheryl P. Gow, Amelia R. Woolums, Murray Jelinski, Stacey Lacoste, Cheryl L. Waldner

**Affiliations:** 1 Department of Large Animal Clinical Sciences, Western College of Veterinary Medicine, University of Saskatchewan, Saskatoon, SK S7N 5B4, Canada; jennifer.abiyounes@usask.ca (J.N.A.Y.);; 2 School of Public Health, University of Alberta, Edmonton, AB T6G 1C9, Canada; simon.otto@ualberta.ca; 3 Canadian Integrated Program for Antimicrobial Resistance Surveillance, Public Health Agency of Canada, Saskatoon, SK S7L 0Z2, Canada; 4 Department of Pathobiology and Population Medicine, College of Veterinary Medicine, Mississippi State University, Mississippi State, MS 39762, USA

**Keywords:** bovine respiratory disease, feedlot, bovine, antimicrobial resistance, antimicrobial use, longitudinal, prevalence, pen, sampling

## Abstract

Antimicrobials are crucial for treating bovine respiratory disease (BRD) in beef feedlots. Evidence is needed to support antimicrobial use (AMU) decisions, particularly in the early part of the feeding period when BRD risk is highest. The study objective was to describe changes in prevalence and antimicrobial susceptibility of BRD bacterial pathogens at feedlot processing (1 day on feed (1DOF)), 12 days later (13DOF), and for a subset at 36DOF following metaphylactic antimicrobial treatment. Mixed-origin steer calves (*n* = 1599) from Western Canada were managed as 16 pens of 100 calves, receiving either tulathromycin (*n* = 1199) or oxytetracycline (*n* = 400) at arrival. Deep nasopharyngeal swabs collected at all time points underwent culture and antimicrobial susceptibility testing (AST). Variability in the pen-level prevalence of bacteria and antimicrobial susceptibility profiles were observed over time, between years, and metaphylaxis options. Susceptibility to most antimicrobials was high, but resistance increased from 1DOF to 13DOF, especially for tetracyclines and macrolides. Simulation results suggested that sampling 20 to 30 calves per pen of 200 reflected the relative pen-level prevalence of the culture and AST outcomes of interest. Pen-level assessment of antimicrobial resistance early in the feeding period can inform the evaluation of AMU protocols and surveillance efforts and support antimicrobial stewardship in animal agriculture.

## 1. Introduction

Antimicrobials are essential for maintaining health and welfare and preventing economic losses in animal production systems. In North American cattle feedlots, parenteral antimicrobials are most frequently used to manage bovine respiratory disease (BRD), the leading cause of morbidity and mortality [1]. While management strategies such as preconditioning, vaccination at arrival, and reducing stress can better prepare cattle for the transition from farm to feedlot, an estimated 39% of calves entering Western Canadian feedlots remain at high risk for developing BRD, and these means alone have been insufficient to adequately manage the disease [2,3,4,5]. As a result, antimicrobials continue to be necessary and have proven effective for BRD control [6].

However, efficient strategies for using laboratory tools to inform antimicrobial use (AMU) decisions for BRD treatment and control are lacking. The need for evidence to target AMU is growing with the global awareness of antimicrobial resistance (AMR), which threatens the efficacy of antimicrobials as well as the health of humans, animals, and the environment [7,8]. Additionally, a better understanding of AMR in common bacterial pathogens in the development of BRD management and treatment protocols is essential for antimicrobial stewardship [9,10]. A reduction in the availability of antimicrobials to treat BRD in feedlot cattle would be detrimental to animal welfare and the productivity of the beef industry [11].

The control and treatment of BRD in feedlots is complicated due to its polymicrobial and multi-etiologic nature. Consistently identified risk factors include the placement of young and lighter-weight calves, vaccination status at arrival, recent and abrupt weaning, prolonged transport time, commingling animals from different origins, and inclement weather [12,13,14]. These stressors are thought to suppress the respiratory immune system, increasing the animal’s susceptibility to contagious pathogens as well as opportunistic viral and bacterial infection, ultimately resulting in respiratory disease. Principal bacterial agents implicated in BRD include three members of the *Pasteurellaceae* family: *Mannheimia haemolytica*, *Pasteurella multocida*, and *Histophilus somni*. Not only have these bacteria been consistently recovered from clinical cases but cattle from which *M. haemolytica* was recovered at arrival were more likely to become ill within 10 days [15].

A 2023 study by Smith et al. [16] collected data from 25 U.S. commercial feed yards, representing 4.4 million cattle on feed, with the objective of determining the temporal distributions of first BRD treatment. The results agree with others showing that BRD incidence is often greatest during the first few weeks on feed [12,17]. However, studies exploring bacterial changes in pathogen and AMR prevalence over time during the early feeding period (<14 days) are only recently gaining interest [18,19,20]. While macrolides are the most effective antibiotics for metaphylaxis to reduce the incidence of BRD, longitudinal changes in bacterial prevalence and AMR after feedlot arrival are less commonly reported than samples collected at arrival [21,22,23]. Consequently, feedlot veterinarians face the challenge of lacking readily available AMR data to inform antimicrobial choices in calves requiring first treatment for BRD. Instead, antimicrobial drug choices are made based on prior experience, animal history, historical treatment records, and historical data on therapeutic effectiveness.

To address the aforementioned challenges related to AMU in feedlots, veterinarians and managers need practical strategies to support laboratory-based antimicrobial decision-making. These strategies should align with the World Health Organization (WHO) recommendations for using laboratory-based tools to select antimicrobials for treatment to promote prudent AMU and monitor AMR in food-producing animals [7]. Commercial feedlots can house thousands of cattle, making it impractical to sample each individual animal due to the associated time, resources, and costs involved. However, as feedlot cattle are managed as groups within pens [6], a subset of animals per pen could be sampled to estimate the frequency of the bacterial pathogens with AMR of interest.

Sampling cattle at feedlot arrival provides insights into incoming levels of AMR, facilitating the effective monitoring, surveillance, and identification of intervention points both pre- and post-arrival. However, factors such as stress, commingling, and environmental contamination lead to modifications in the upper respiratory microbiome and affect the prevalence of bacteria and AMR after arrival [24,25]. Additionally, cattle at a high risk of developing BRD are typically administered metaphylactic antimicrobial therapy within the first few days of feedlot arrival to decrease the pathogen burden and reduce the risk of pen-level disease [6]. Metaphylaxis may induce selective pressure and result in greater proportions of AMR bacteria [26,27,28,29,30], with differences in the nasopharyngeal microbiota of treated cattle up to 60 days on feed [23,31]. Thus, in cattle administered long-acting injectable antimicrobials for metaphylaxis, sampling after a post-metaphylactic interval (PMI) could provide more meaningful data on the pathogen and AMR prevalence that occurs during the early feeding period with the greatest BRD risk, and could better inform future antimicrobial treatment decisions.

The overall goal of this study was to leverage an opportunity to sample all calves from auction-sourced feedlot pens at 1 and 13 days on feed (DOF) to determine the prevalence of selected BRD pathogens and clinically relevant AMR targets. This study is unique because of the large number of calves (*n* = 1599) sampled over a two-year period [19,20,22]. Calves in each purchased lot were auction-sourced from multiple farms and housed in pens of 100 head, which more closely approximated commercial feedlot practices and comingling risk in Western Canada compared to pen sizes from previous reports [19,20,32,33]. Complete culture and antimicrobial susceptibility data from all calves in larger pens at two consistent time points across different years and metaphylaxis protocols expand on other studies evaluating changes in the first few weeks on feed [19,22,34].

The first objective was to describe the prevalence and variability of BRD pathogens and AMR among pens, metaphylaxis groups, and years during the early feeding period, including 1DOF, 13DOF, and 36DOF, for fall-placed auction market calves at Western Canadian feedlots. The second objective was to compare the prevalence of BRD pathogens and associated AMR recovered from calves at 1DOF with those observed at 13DOF to evaluate the additional insights gained from sampling following the PMI.

## 2. Results

### 2.1. Study Population

Radio Frequency Identification (RFID) tag numbers were scanned for each calf. In 2020, calves were sourced from 292 unique herds of origin (derived from the first 12 digits of the calves’ 15-digit RFID tag) (as described in Section 4.2) (Table 1). The number of unique herds of origin for 2020 calves purchased for the feedlot varied from 30–81 herds per pen, suggesting a high risk of pathogen exposure due to commingling. The calves sampled in 2021 were less diverse, with 208 unique herds of origin and 12–38 unique herds per pen. At 1DOF, the mean calf weight in 2020 was 253 kg (556 lbs) (range: 211–291 kg (464–640 lbs)) (Table 1). Lighter calves were targeted in 2021, resulting in a mean calf weight of 225 kg (496 lbs) (range: 160–315 kg (351–694 lbs)).

Of the 1600 steers purchased for this study, one calf was recumbent at the time of initial feedlot processing (as outlined in Section 4.3) in 2020 and removed, resulting in 1599 cattle sampled at 1DOF from October to December. Three calves died prior to 13DOF (one calf in November of 2020 and two in November of 2021), leaving a total of 1596 calves sampled at 13DOF. At 36DOF, 310 calves were sampled. Antimicrobial susceptibility data were not available from the laboratory for four samples in 2021 (two at 1DOF, one at 13DOF, one at 36DOF), resulting in the following total samples cultured and tested for susceptibility: 1597 at 1DOF, 1595 at 13DOF, and 309 at 36DOF.

For all pens combined, 8.1% of calves (*n* = 130) were treated for BRD within 45DOF. Cohorts receiving tulathromycin metaphylaxis observed similar BRD incidences, with 3.5% (*n* = 28) of calves treated in 2020 and 3.3% (13 calves) treated in 2021 for tulathromycin-treated cohorts. In contrast, in 2021, oxytetracycline-treated cohorts observed a higher incidence of 22.3% of calves (*n* = 89) being treated for BRD. Total mortalities in 2020 included three calves (0.4%) succumbing to BRD and one case of bloat. In 2021, mortality attributed to BRD increased slightly to 1.0% (eight calves), with 0.6% (five calves) from pen 16. Other mortalities in 2021 included four cases of bloat (0.5%) and one calf (0.1%) euthanized due to neurologic symptoms that also had lung lesions.

### 2.2. Differences in Bacterial Recovery between Years and Metaphylaxis Options

The proportion of calves that were culture-positive for bacteria of interest (*M. haemolytica*, *P. multocida*, and *H. somni*) varied among pens during the study (1DOF, 13DOF, and 36DOF) and between sampling years (Table 2 and Table 3).

**Figure 1:**
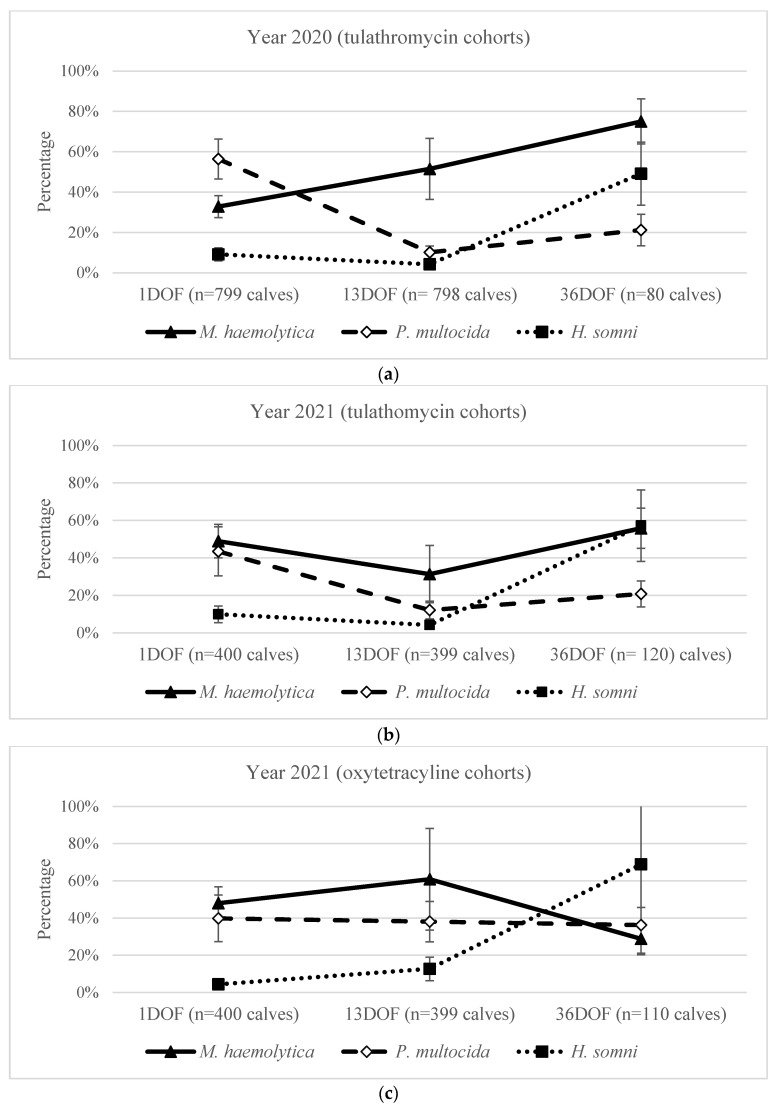
Percentage (%) of calves from which *M. haemolytica*, *P. multocida*, and/or *H. somni* were recovered at 1DOF, 13DOF, and 36DOF for (**a**) 2020 pen cohorts that received tulathromycin metaphylaxis, (**b**) 2021 pen cohorts that received tulathromycin metaphylaxis, and (**c**) 2021 pen cohorts that received oxytetracycline metaphylaxis. Samples at 1DOF were collected during on-arrival processing and before metaphylaxis. Percentages displayed are population-averaged with 95% confidence intervals, adjusted for clustering at the pen level using mixed effects logistic regression. For pen 16, sampling at 30DOF, prior to mass treatment, was used for analysis.

Irrespective of year, for all calves sampled at 1DOF, prior to the administration of metaphylactic antimicrobials, 41% (CI: 35–46%) were culture-positive for *M. haemolytica*, 49% (CI: 41–57%) for *P. multocida*, and 8% (CI: 6–11%) for *H. somni* (Figure 1a–c). However, the proportion of calves that were culture-positive for *M. haemolytica* at 1DOF was higher in 2021 (49% of calves [CI: 42–55%]) compared to 2020 (33% of calves [CI: 27–39%]) (Table 4). In contrast, *P. multocida* was recovered from more calves in 2020 (56% [CI: 46–66%]) than in 2021 (42% [CI: 32–52%]) at 1DOF. The recovery of *H. somni* at 1DOF did not differ between 2020 (9% [CI: 5–14%]) and 2021 (7% [CI: 4–11%]).

When adjusting for year, metaphylaxis antimicrobial, and clustering at the pen level, there were no significant differences in the recovery of *M. haemolytica* at 13DOF across sampling years and metaphylactic treatment groups (Table 5). *P. multocida* was around five times more likely to be cultured from calves at 13DOF that had received metaphylactic oxytetracycline in 2021 compared to calves from either year that received tulathromycin (Table 5). For calves that received tulathromycin on arrival, there was no difference (*p* = 0.55) in *P. multocida* recovery at 13DOF between years (Table 5). *H. somni* was 3.3 times more likely to be cultured at 13DOF from calves in 2021 treated with oxytetracycline at arrival than calves in 2020 treated with tulathromycin (Table 5). There was no difference in the recovery of *H. somni* at 13DOF between 2020 and 2021 tulathromycin-treated calves (*p*-value: 0.06) or between year 2021 tulathromycin- and oxytetracycline-treated calves (*p*-value: 0.67) (Table 5).

### 2.3. Differences in Bacterial Recovery over Time within Year and Metaphylaxis Options

The probability of recovering *M. haemolytica* more than doubled from 33% (CI: 27–39%) at 1DOF before metaphylaxis to 75% (CI: 64–84%) at 36DOF post metaphylaxis administration in 2020 (Figure 1a). While there was substantial pen-to-pen variation at 13DOF (Table 2), calves in 2020 were more likely to have *M. haemolytica* isolated at 13DOF than at 1DOF and at 36DOF compared to 13DOF (Table 6).

In contrast, the recovery of *M. haemolytica* from pens receiving tulathromycin in 2021 decreased from 1DOF to 13DOF (49% [CI: 40–58%] to 31% [CI: 16–52]), followed by an increase from 13DOF to 36DOF (Figure 1b; Table 6). For calves receiving oxytetracycline at arrival in 2021, the recovery of *M. haemolytica* decreased from 1DOF to 36DOF (Figure 1c; Table 6).

For calves receiving tulathromycin at arrival, *P. multocida* recovery decreased between 1 and 13DOF from 56% (CI: 46–66%) to 10% (CI: 7–14%) in 2020 and from 44% (CI: 30–58%) to 12% (CI: 7–19%) in 2021 (Figure 1a, b; Table 6). *P. multocida* then increased in the tulathromycin groups from 13DOF to 36DOF in both 2020 and 2021 (Figure 1a, b; Table 6). In contrast, the recovery of *P. multocida* from calves receiving oxytetracycline at arrival in 2021 did not significantly change between 1DOF (40% [CI: 27–54%]) and 36DOF (36% [CI: 27–47%]) (Figure 1c; Table 6).

*H. somni* recovery increased over time, averaging 8% at 1DOF and 13DOF and sharply increasing to 58% at 36DOF (Figure 1a–c). However, recovery varied from 1DOF to 13DOF across metaphylaxis options (Table 6). In year 2020 tulathromycin-treated pens, calves were less likely to have *H. somni* at 13DOF than at 1DOF. In 2021, there was no significant difference for tulathromycin-treated calves; however, the recovery of *H. somni* was greater in oxytetracycline-treated calves at 13DOF (Table 6).

### 2.4. Bacterial Co-Isolation Patterns in Years 2020 and 2021 at 1DOF and 13DOF

Most calves with BRD bacteria detected had a single species isolated at both 1DOF and 13DOF (Table 7). Irrespective of year and metaphylaxis antimicrobial administered, the most common co-isolation pattern observed at 1DOF was *M. haemolytica* and *P. multocida* (15%), followed by *P. multocida* and *H. somni* (3%) and *M. haemolytica* and *H. somni* (0.9%) (Table 7). At 13DOF, these proportions changed only slightly, with 8% of calves having *M. haemolytica* and *P. multocida*, 2% having *M. haemolytica* and *H. somni*, and 1% having *P. multocida* and *H. somni*. Very few calves had all three bacteria isolated concurrently.

In general, calves that received metaphylaxis treatment with tulathromycin had a substantial decrease in overall bacterial recovery from 1DOF to 13DOF (OR: 2.91; 95% CI: 2.45–3.47; *p*-value: <0.001), with an average of 22% of calves with a negative culture result at 1DOF and 45% at 13DOF (Table 7). Within this overall pattern, the recovery of *M. haemolytica* increased between 1DOF and 13DOF in 2020, although not in 2021 (Figure 1). This contrasted with the results for oxytetracycline-treated calves, for which the average number of animals with negative culture results remained relatively stable over time (OR: 0.77; 95% CI: 0.56, 1.08; *p*-value: 0.13) (Table 7).

### 2.5. Differences in Antimicrobial Susceptibility of Bacteria between Years at 1DOF

The overall crude (unadjusted) prevalence of calves exhibiting resistance to the tested antimicrobials among the BRD pathogens of interest at 1DOF was low (Table 8 and Table 9). The population-averaged prevalence, accounting for clustering by pen, was 7% (CI: 5–10%) for 2020 and 5% (CI: 4–8%) for 2021 (*p*-value: 0.23). Frequency tables for minimum inhibitory concentrations are provided in Supplementary Materials S1.

In 2020 at 1DOF, the most commonly observed resistance was to tetracycline (4% of calves) or spectinomycin (4% of calves) with *P. multocida* (Table 8). For all three species, <2% of calves had isolates resistant to ampicillin, tulathromycin, gamithromycin, or tildipirosin. No calves had bacteria with resistance to penicillin, ceftiofur, danofloxacin, enrofloxacin, florfenicol, or tilmicosin.

In 2021 at 1DOF, 2% of calves had ampicillin- or spectinomycin-resistant *P. multocida* (Table 9). Tetracycline resistance was observed in 0.8% of calves in the tulathromycin-treated cohorts and 2% in the oxytetracycline-treated cohorts. Less than 1% of calves had *M. haemolytica* or *H. somni* with any AMR. No calves had bacteria with resistance to enrofloxacin, florfenicol, or tulathromycin.

### 2.6. Differences in Antimicrobial Susceptibility of Bacteria between Years at 13DOF

The population-averaged prevalence of calves with an organism resistant to at least one antimicrobial at 13DOF, adjusted for pen, was 41% (CI: 23–62%) for year 2020 tulathromycin-treated calves, 15% (CI: 5–37%) for 2021 tulathromycin-treated calves, and 20% (CI: 7–46%) for 2021 oxytetracycline-treated calves. There were no significant differences in the prevalence of calves with at least one AMR pathogen at 13DOF between year/metaphylaxis groups (2021/tulathromycin vs. 2020/tulathromycin, *p*-value: 0.062; 2021/oxytetracycline vs. 2020/tulathromycin, *p*-value: 0.18; 2021/tulathromycin vs. 2021/oxytetracycline, *p*-value: 0.65).

The highest prevalence of AMR was observed for tulathromycin- or gamithromycin-resistant *M. haemolytica* isolated from calves in 2020 at 13DOF (Table 10). Calves were more likely to have tulathromycin- or gamithromycin-resistant *M. haemolytica* isolates from 2020 tulathromycin-treated pens than either 2021 tulathromycin-treated or 2021 oxytetracycline-treated pens (Table 10). There were no significant differences between years for the frequency of recovery of tilmicosin-, tildipirosin-, or tetracycline-resistant *M. haemolytica* (*p*-values of 0.36, 0.20, and 0.52, respectively).

Tetracycline-resistant *P. multocida* was not identified at 13DOF in any calves in the 2021 tulathromycin-treated pens, compared to 13% with tetracycline-resistant isolates in the 2021 oxytetracycline-treated calves at 13DOF (Table 9). Tetracycline-resistant *P. multocida* was more prevalent at 13DOF in 2021 oxytetracycline-treated calves than either the 2020 or 2021 tulathromycin-treated calves (Table 10).

### 2.7. Within-Year Comparison of Bacterial and Antimicrobial Susceptibility from 1DOF to 36DOF

For the 2020 study population, the population-averaged prevalence of tulathromycin-resistant *M. haemolytica* increased over time from 0.1% (CI: 0.02–0.9%) at 1DOF to 34% (CI: 17–57%) at 13DOF (*p*-value: <0.001; Table 8 and Table 11). The prevalence of calves with gamithromycin-resistant *M. haemolytica* also increased from 0.1% (CI: 0.02–0.9) at 1DOF to 35% (CI: 17–58) at 13DOF (*p*-value: <0.001; Table 8 and Table 11). Both tulathromycin- and gamithromycin-resistant *M. haemolytica* also increased from 1DOF to 36DOF (Table 11). There were no significant differences over time for tildipirosin-resistant *M. haemolytica*, while resistance to tilmicosin increased across all time points (Table 11).

In 2021, there were no calves with tulathromycin- or gamithromycin-resistant *M. haemolytica* at 1DOF (CI: 0–0.005%) in either metaphylaxis group (Table 9). By 13DOF, the population-averaged prevalence of tulathromycin-resistant *M. haemolytica* in the year 2021 increased to 7% (CI: 2–22%) for tulathromycin-treated calves but only to 0.8% (CI: 0.5–15%) for oxytetracycline-treated calves (Table 11). In 2021 tulathromycin-treated cohorts, gamithromycin-, tildipirosin-, and tilmicosin-resistant *M. haemolytica* increased from 1DOF to 13DOF and from 1DOF to 36DOF, but the difference between 13DOF and 36DOF was not significant. In contrast, there were no significant differences over time for the oxytetracycline-treated cohorts (Table 11).

Tetracycline-resistant *M. haemolytica* also increased in 2021 tulathromycin-treated calves across all time points (Table 11), but there were no significant differences for the oxytetracycline-treated cohorts. The recovery of *P. multocida* with tetracycline resistance decreased from 1DOF to 13DOF in 2020 (Table 12) but subsequently rebounded from 13DOF to 36DOF (*p*-value: 0.05). There were no differences over time in 2021 tulathromycin-treated calves. For oxytetracycline-treated calves in 2021, the recovery of tetracycline-resistant *P. multocida* increased significantly from 1DOF to 13DOF as well as from 1DOF to 36DOF, with no change between 36DOF and 13DOF (Table 12).

### 2.8. Pen-Level Clustering of Bacterial Recovery and Antimicrobial Resistance

For all calves at 1DOF, differences in the recovery of bacteria of interest between pens measured by the intraclass correlation coefficient (ICC) were low at 0.029 (CI: 0.011–0.078) for *M. haemolytica*, 0.09 (CI: 0.04–0.18) for *P. multocida*, and 0.10 (CI: 0.04–0.23) for *H. somni*. In comparison, the proportion of variance explained by clustering at the pen level increased substantially for the recovery of *M. haemolytica* at 13DOF to 0.24 (CI: 0.13–0.39). The ICCs for *P. multocida* at 13DOF (0.067, CI: 0.025–0.16) and *H. somni* (0.16, CI: 0.06–0.35) were relatively stable compared to 1DOF.

Likewise, the prevalence of tulathromycin-resistant *M. haemolytica* was low (0.1%) at 1DOF, making the ICC negligible. At 13DOF, while conditioning on year and metaphylactic drug, the proportion of total variance explained by pen increased substantially to 0.72 (CI: 0.46–0.89). A similar increase in pen-level variation was observed for calves with tetracycline-resistant *M. haemolytica* where, again, the ICC was negligible at 1DOF but increased to 0.74 (CI: 0.29–0.95) at 13DOF. For calves with tetracycline-resistant *P. multocida*, the ICC at 1DOF was 0.078 (CI: 0.012–0.35), increasing to 0.39 at 13DOF (CI: 0.16–0.68).

### 2.9. Sample Size Estimates Generated from Simulation Models

The accuracy and precision of sample size estimates resulting from simulation studies are detailed in Supplementary Materials S2, Figure S1. The findings support the recommendation of sampling 20 to 30 calves per pen of 200 calves. This sample size range enabled the differentiation of a low, moderate, or high prevalence of calves with BRD pathogens exhibiting antimicrobial resistance.

## 3. Discussion

The results of this study support the antimicrobial stewardship efforts of the beef feedlot industry by providing evidence of pathogen and AMR variability over time and between pens. Currently, feedlot cattle are managed in groups at the pen level and antimicrobial decisions are based on experience, available history of the incoming cattle, studies on protocol effectiveness, and limited AMR surveillance reports. Sampling each individual animal prior to AMU might be an ultimate end goal of targeted antimicrobial decisions [7]; yet, it is currently neither feasible nor practical for large commercial feedlots. Instead, our simulation model supports that evidence-based laboratory data on individual pens could be generated by sampling a subset of 20 to 30 animals per pen of 200 calves at arrival for cattle not receiving metaphylaxis or shortly after the PMI in calves where metaphylaxis was used. The resulting culture and antimicrobial susceptibility testing (AST) data could then be used to inform pen-level management decisions and antimicrobial treatment protocols for animals that become sick after testing. Despite limitations in laboratory testing data and cut points with which to interpret them, antimicrobial stewardship can be promoted by reducing the use of antimicrobials likely to be ineffective based on their resistance profiles. Providing laboratory data that AMU is evidence-based can also address the demands of stakeholders, trade partners, and consumers who are increasingly concerned with the use of antimicrobials in animal agriculture [35,36].

Identifying optimal sampling times used to inform AMU decisions requires a comprehensive understanding of bacterial dynamics and antimicrobial susceptibility changes within feedlot pens during the early feeding period, when calves are at increased risk of BRD. This study therefore focused on sampling cattle at arrival, and prior to metaphylaxis treatment and prolonged comingling with pen mates, then again two weeks after metaphylaxis. As such, this study provides foundational knowledge needed for subsequent studies to build upon.

The design of this study was distinct from and built on findings from others evaluating changes in the early feeding period in several ways. First, multiple samples were collected from almost 800 calves per year and the study was repeated over the course of two years and with different metaphylaxis protocols to provide robust data for description and comparison. Second, animals in this study were sourced from mixed farms of origin (30–81 different farms per pen in 2020 and 12–38 farms per pen in 2021) and housed in pens with calf numbers that more closely approach those observed in commercial feedlots than pen sizes typically reported in research studies. As a result, this information builds on the work of other longitudinal research investigations that have used smaller pen numbers (20–30 calves/pen) [18,19] or enrolled cattle with limited diversity in herd of origin [19,24]. Perhaps most distinctively, this study maintained a consistent sampling approach, collecting a sample from each animal at two specific time points, with all samples submitted for AST. This contrasts with other studies that only sampled a subset of animals from commercial feedlot pens [15] in which not all recovered bacteria underwent AST [37] or the second sampling times occurred at varying DOF [15,38,39]. Our study’s second time point of 13DOF provided further data on bacterial changes during the first two weeks on feed as opposed to others focusing on the evolution of bacteria from arrival to ≥40DOF [20,24,40,41] or differences between bacterial isolation in auction-derived and ranch-direct calves [18].

While sampling cattle at 1DOF captures baseline pathogen and AMR statuses at feedlot entry, the 13DOF time point is significant for its insight into shifts in bacterial prevalence and antimicrobial susceptibility following feedlot placement. The first few weeks on feed are an important time in the feedlot for the development of clinical BRD and the transmission and dissemination of BRD pathogens and AMR within calves in pens and between pens [31,42]. Commingling of animals from different sources, stress, potential environmental contamination, exposure to fomites, changes in diet, and metaphylactic antimicrobial administration can each affect respiratory microflora [31,43]. In this study, the choice of 13DOF sampling coincides with the maximum PMI for tulathromycin [44] and the period when pathogen numbers might rebound, and any AMR-associated selection from AMU at arrival or transmission might emerge.

Although national surveillance programs support efforts to monitor BRD pathogens and AMR trends over time, allowing for prevalence comparisons between geographies, these programs generally collect samples from a subset of animals from a subset of pens to estimate commodity- or feedlot-level prevalence [45,46]. To enhance compliance, the samples for the surveillance program are collected when it is most convenient for feedlot staff, at arrival and reprocessing. In contrast, the purposeful design of the present study repeatedly sampled all animals within each pen across the two time points early in the feeding period, which is of great interest for potentially informing the treatment of BRD.

In the present study, *P. multocida* was the most commonly recovered bacteria at arrival in 2020, while *M. haemolytica* was more common at arrival in 2021 and remained the prominent bacteria recovered at 13DOF for both years. This contrasts with the recent studies by Nobrega et al. [22] and Guo et al. [40], where *P. multocida* was reported as the most commonly recovered bacteria at arrival and throughout most subsequent time points. Although both studies noted above agreed in describing changes in pathogen recovery within calf groups over time [22,40], the present study was unique in further evaluating the specific effect of the group-level clustering of outcomes.

While no formal assessments of group effects could be made due to the size of the study, a previous report did describe differences in AMR across groups [18]. Hirsch et al. [18] compared the presence of bacterial pathogens and AMR from two groups of 30 cattle either directly transported to a feedlot or first transported to an auction market prior to feedlot placement. Deep nasal swabs were collected at feedlot processing (e.g., on arrival), 2DOF, and 9DOF [18]. *P. multocida* was also the most frequently isolated bacteria at the time of feedlot processing in their study, regardless of transport group. While their objective was to compare sampling times between auction market and ranch-direct calves, differences were noted in the prevalence of bacteria over time between the two feedlot groups, with one group experiencing the spread of a multi-drug-resistant strain of *P. multocida* while the second group observed no recovery of *P. multocida* at 9DOF or 30DOF [18]. Together, these results demonstrate the potential variation in bacterial behavior over time observed across different populations of animals enrolled within the same study.

The percentage of calves from which the organisms of interest were recovered was generally higher in this study compared to others, particularly for *M. haemolytica* [15,34,38]. The *H. somni* trends were similar to those observed by Erickson et al. [38] during earlier time points, but prevalence was higher at later sampling times. The on-arrival prevalence of bacteria was also higher than the first year of national surveillance averages reported by the Canadian Integrated Program for Antimicrobial Resistance Surveillance (CIPARS) [47]. The CIPARS project observed a 2020 national on-arrival isolate recovery rate of 8.9% (34/384 samples) for *M. haemolytica*, 28% (108/384 samples) for *P. multocida*, and 2.6% (10/384 samples) for *H. somni* (Sheryl Gow, personal communication). In comparison, from all years combined, the present study observed a crude on-arrival recovery of 41% of calves with *M. haemolytica*, 49% with *P. multocida*, and 8% with *H. somni*.

The current study adhered to the identical protocol for bacterial culture and AST performed at the same diagnostic laboratory utilized by CIPARS. One possible reason for the discrepancies in recovery rates between this study and CIPARS is the differences in the risk levels of the cattle sampled. Surveillance by CIPARS collected samples from a range of risk categories and was not restricted to the fall season, whereas the present study focused on fall-placed auction-sourced calves. Moreover, the samples in the current study had the advantage of a fast turn-around time from sample collection to arrival and processing at the laboratory. This delay was a few hours for the present study compared to days for CIPARS samples collected and shipped by courier from commercial feedlots.

The present study reports a higher proportion of calves with *M. haemolytica* with resistance to gamithromycin and tulathromycin than to tilmicosin or tildipirosin in 2020, but the results from 2021 are more consistent with the CIPARS surveillance report for 2019 [47]. Both our study and the 2019 CIPARS report identified tetracycline as the antibiotic with the highest resistance prevalence for *P. multocida*, while *H. somni* exhibited the least amount of AMR among the three bacteria [47]. Overall, the present study agreed with CIPARS in observing low AMR on feedlot arrival, with subsequent increases noted at second sampling times. A direct comparison of resistance prevalence between the two studies should be conducted cautiously due to differences in reporting: CIPARS presents resistance prevalence at the per-isolate level, whereas our study reports at the per-calf level in the primary text. However, isolate-level information in the present study can be derived from the MIC tables available in the Supplementary Materials S1.

In the present study, the on-arrival resistance of *M. haemolytica* isolates was lower than that reported by Andrés-Lasheras et al. [34] in their longitudinal study using 10 commercial Alberta feedlots. Andrés-Lasheras et al. [34] reported that their most common on-arrival AMR resistance in *M. haemolytica* isolates collected from beef-type cattle included oxytetracycline (10%), tilmicosin (6.4%), and ampicillin (4.6%). In comparison, in the present study, only one oxytetracycline- (0.2%), two tilmicosin- (0.3%), and three ampicillin-resistant *M. haemolytica* (0.5%) were recovered from cattle on arrival. The resistance of *P. multocida* isolates was also slightly higher in the Andrés-Lasheras et al. study [34], with their most frequent resistance profiles being for tetracycline (8.4%), ampicillin (7.7%), and spectinomycin (8.1%), compared to recoveries in the present study for tetracycline (5.1%), ampicillin (4.1%), and spectinomycin (6.0%). *H. somni* consistently exhibited minimal AMR on arrival and was observed at similar levels in the present study, the study by Andrés-Lasheras et al., and CIPARS [34].

Antimicrobial exposure contributes to changes in microbiota [23,31]. Metaphylaxis is an important strategy for BRD control in high-risk calves that results in an alteration in pathogen load [3,6]. However, a likely secondary consequence of blanket AMU is a change in the antimicrobial susceptibility patterns of bacteria, even if transient. This phenomenon was observed in the pens studied, where select antimicrobial MIC distributions trended up from 1DOF to 13DOF and generally decreased again by 36DOF first in 2020 and then again in 2021 [48]. This shift in susceptibility distribution differed between pens and could have been missed if pens had only been sampled at arrival and later in the feeding period (>40DOF). Other longitudinal studies have also observed an increase in MIC values in respiratory bacteria over the feeding period [10,15,22,26].

The results of this study are also consistent with evidence of the dissemination of strains of BRD pathogens containing AMR among feedlot calves [18,40,49,50]. The rapid increase in macrolide-resistant *M. haemolytica* in calves within pens and the consistency in this pattern across five of eight pens of the 2020 study population was unique compared to what was observed in either metaphylaxis treatment groups from 2021. A more thorough investigation of the changes in phenotypic susceptibility of *M. haemolytica* as demonstrated by MIC results observed during the early feeding period in the year 2020 has previously been described [48]. In agreement with the present study, Guo et al. [40] also found that while the respiratory microbiota of beef calves can increase in diversity from calf ranch to feedlot, the results also vary by calf ranch and feedlot.

Also in agreement, Hirsch et al. [18] also noted bacterial and antimicrobial susceptibility variations in feedlot calves. They compared two groups, one directly transported to a feedlot and the other to an auction market before feedlot placement. Deep nasal swabs were collected at feedlot processing (e.g., on-arrival), 2DOF, and 9DOF [18]. While their objective was to compare sampling times between auction market and ranch-direct calves, differences were noted in the prevalence of bacteria recovered over time between the two feedlot groups, with one group experiencing the spread of a multi-drug-resistant strain of *P. multocida* while the second group observed no recovery of *P. multocida* at 9DOF or 30DOF [18]. Together with our study, these outcomes demonstrate the potential variability in bacterial behavior across different animal populations, reinforcing the importance of pen-level sampling.

The proportion of calves with bacteria resistant to the antibiotic class used for metaphylaxis increased between arrival processing and the second sample at 13DOF. Additionally, macrolide-treated pens in 2020 showed evidence of the clonal spread of macrolide-resistant *M. haemolytica* [48]. Our findings correspond with those of other studies on feedlots linking antimicrobial use to the selection of oxytetracycline-resistant *P. multocida* [40] and antimicrobial-resistant *M. haemolytica* clones [49,50]. Studies by Woolums et al., Snyder et al., and Crosby et al. also revealed a high prevalence of macrolide-resistant and multi-drug-resistant (MDR) *M. haemolytica* in stocker cattle post metaphylaxis treatment with macrolides [27,28,29].

Furthermore, Holman et al. [31] described significant alterations in nasopharyngeal microbiota due to single administrations of either oxytetracycline or tulathromycin metaphylaxis treatments in commercial feedlots. An association between oxytetracycline metaphylaxis and AMR was also evident, specifically shown by a significant increase in tet(H), a gene responsible for tetracycline efflux, observed from entry to exit of the feedlot period.

While recent studies suggest a trend between antimicrobial classes used in metaphylaxis and subsequent AMR patterns, inconsistencies exist. For instance, a longitudinal study of feedlot cattle by Nobrega et al. [22] found no association between tetracycline metaphylaxis and tetracycline MICs in respiratory bacteria. However, they did note higher MICs for macrolides after parenteral metaphylaxis treatment.

Moreover, Woolums et al. [28] highlighted that, despite receiving metaphylaxis treatment with tildipirosin on arrival, all *M. haemolytica* isolates from their group of stocker cattle were resistant to enrofloxacin prior to exposure to the fluoroquinolone antimicrobial class. This suggests that antimicrobial exposure might provide advantages to different resistant strains beyond the administered antimicrobial class, raising concerns about the unpredictable effects of such exposure [28]. A better understanding of the drivers of AMR, whether from selective pressure or the dissemination of resistant clones, is an area deserving of future research.

In addition to the potential to initiate resistance to antimicrobials beyond those used in treatment, AMU can also affect non-target bacterial populations. This was demonstrated in a longitudinal study by Holman et al. [23] that evaluated the effects of oxytetracycline and tulathromycin on the fecal and nasopharyngeal microbiota of feedlot cattle. Both antibiotics altered fecal and nasopharyngeal microbiota, highlighting AMU’s broader impact. While antimicrobials are vital for BRD treatment, the collateral effects of disrupting the antimicrobial susceptibility of several bacterial species underscores the need for precise drug selection. This is essential to mitigate potential cost–benefit implications of AMR emergence in non-target bacterial populations.

The differences in the prevalence of both BRD bacteria and AMR observed at 1DOF and 13DOF in this study raise questions regarding the most appropriate time point for sampling feedlot calves within pens. As is often the case with complex systems, there is no one-size-fits-all answer, and sampling times will depend on the reason for action. There are limited publications directly comparing the temporality of BRD in cattle receiving on-arrival antimicrobials compared to cattle that do not. Older studies performed prior to the consistent use of metaphylaxis that examined the timing of BRD have reported a high incidence of disease during the first weeks on feed [14,51]. Therefore, sampling cattle on arrival can provide data on the bacteria and antimicrobial susceptibility profiles of incoming cattle and could be useful in situations where metaphylaxis is not used and calves are at risk of BRD treatment within the first few DOF. On-arrival sampling strategies would also benefit AMR surveillance efforts in monitoring AMR risk based on exposure prior to feedlot entry and targeting critical intervention points. This, in turn, would contribute to proactive mitigation strategies for AMR surveillance that are important for human and animal health.

However, sampling cattle at a single time point only provides a snapshot in time and, while useful for directing immediate therapeutic decisions in animals that fall ill shortly after sampling, this is insufficient for predicting the development and directionality of future AMR dynamics. Further, the low frequency of AMR bacteria on arrival and rapid evolution of bacterial communities in the early feed period suggests that sampling after an antimicrobial’s PMI might prove more useful for informing subsequent treatment decisions than samples collected at arrival.

Animals in food production systems are aggregated and managed in groups. This hierarchical structure results in cattle within the same feedlot pen being more like each other and experiencing similar exposures to infectious disease than cattle in different pens. To the authors’ knowledge, no other studies have specifically reported this effect of the clustering of cattle within pens on outcomes of interest related to BRD pathogen recovery and subsequent AMR by calculating the ICC. An ICC value of zero would imply no correlation in observations for calves within their cluster (i.e., pen), while an ICC value of one would indicate identical observations for calves within the same cluster [52]. In this study, the ICC was low for all outcomes analyzed at 1DOF, which was to be expected as the animals had been aggregated into their respective groups for less than 24 h. However, by 13DOF, ICCs increased substantially for *M. haemolytica* as well as for tulathromycin- and tetracycline-resistant *M. haemolytica*, implying high variability in the prevalence of resistance between pens. ICC estimates can vary between studies and populations and are important for understanding the effect of pen-level clustering [53].

Overall, this study provides support for practical, pen-level sampling. The explorations made lay the foundation necessary to begin building strategies for informed and justifiable antimicrobial treatment choices in BRD calves. While unanswered questions remain, fundamental aspects needed to be addressed regarding the need for pen-level sampling and were necessary first steps for future advancements in this realm.

### Study Limitations

All samples were collected by two previously trained individuals in a facility with a hydraulic chute and neck extender with good restraint. Further in this study, an aliquot from a pooled sample of three DNP swabs per animal was used for bacterial culture. This technique might have improved the likelihood of obtaining a sample that represented the colonization status of the nasopharynx of the animal and increased the likelihood of a positive culture result in this study compared to those from which only one DNP was utilized [54,55,56]. Additionally, sample handling and transport time can affect the viability of bacteria prior to arrival at the laboratory. Samples were delivered to the diagnostic laboratory in less than an hour following sample collection from the last calf. Thus, direct comparisons between recovery rates of studies could be impacted by transport times.

The use of DNP swabs is also limited with respect to reflecting pathogens of the lower respiratory tract responsible for BRD. However, existing studies suggest reasonable agreement between upper and lower respiratory tract sampling techniques [54,57,58,59,60], despite the biological variability [61]. Regardless, DNP swabs are more easily implemented into commercial feedlot settings compared to more time-consuming and technically challenging sampling options such as transtracheal wash or bronchoalveolar lavage [62].

Bacterial culture has limited sensitivity, despite its frequent use as a gold standard [63]. The present investigation selected one isolate of each organism for AST. While this is a conventional practice used in part to reduce the costs associated with laboratory testing, the method assumes that all colonies within a culture plate display a common AMR pattern [22,34,47]. The number of colonies needed to estimate the diversity of isolates present on a primary culture plate has been determined for other bacterial pathogens [64]. To investigate this question in reference to *M. haemolytica* and describe the potential phenotypic and genotypic diversity of *M. haemolytica* isolates from individual animals, Carter et al. collected DNP swabs from 28 cattle at risk of, or treated for, BRD [65]. Up to 20 *M. haemolytica* colonies were selected per plate (up to 100 colonies per nasopharyngeal swab) [65]. Using a previously established genotyping technique [66], the study found *M. haemolytica* isolates from individual calf samples to be uniform in both genotype and AMR phenotype and suggested that the selection of few colonies could sufficiently represent the relevant susceptibility pattern of the plate [65]. In contrast, the use of pulse field gel electrophoresis (PFGE) led to the finding that calves can have more than one cluster of *M. haemolytica* when multiple isolates are taken from a plate, even if most were identical [67]. Another study also identified that calves have the potential to simultaneously shed *P. multocida* isolates with differing plasmid profiles [30]. Together, these studies indicate that while a dominant strain of bacteria might exist within a sample, the present study could have missed resistant isolates if only one colony per plate was selected for testing, particularly in the case of *H. somni*, for which the diversity of isolates within individual plates has not been evaluated [65,67].

In addition to the bacteria belonging to the *Pasteurellaceae* family, *Mycoplasma bovis* is another bacterium implicated in BRD, particularly in chronically ill animals [68]. While *M. bovis* was investigated, the approach to culture and MIC data followed a distinct protocol that was not directly comparable to the methodology applied for the other three bacteria of interest. As such, *M. bovis* data will be examined in a subsequent report.

Commercial feedlots are described as having BRD morbidity rates of 10–30% in auction-derived calves, and mortality rates for animals treated for BRD are posited to be around 5–10% [69]. In contrast, the present study observed a relatively low number of calves receiving first treatment for BRD, with an average of 8.1%. The BRD mortality rate was also low at 0.7% and not all of these animals had received prior BRD treatment. The smaller pen size of 100 cattle in our study might have played a role in contributing to the comparatively lower rates when contrasted with commercial feedlots that accommodate 150–300 cattle per pen, with total holding capacities ranging from 15,000 to 25,000. A higher morbidity rate would likely have led to increased AMU, potentially leading to a greater prevalence of AMR over time or a greater diversity in resistance more comparable to commercial feedlots. Most studies of AMR in BRD pathogens in feedlot cattle have not been designed to assess the specific impact of phenotypic AMR on BRD outcomes [70], which is an important area for future research.

## 4. Materials and Methods

### 4.1. Ethical Statement

The research protocol was approved by the University of Saskatchewan Animal Care Committee (AUP 20190069).

### 4.2. Study Population

Recently weaned steers of various beef breeds were sourced from a regional auction market in Saskatchewan, typical of Western Canada. One hundred calves were purchased once a week for 8 weeks in 2020 and again in 2021. Placements occurred in the fall from 6 October to 1 December 2020 and 28 September to 16 November 2021. Each group of 100 calves was maintained as a single cohort and assigned to consecutive feedlot pens. Herd of origin was approximated using the first 12 digits of the calves’ 15-digit RFID tags. Calves from the same herd of origin have unique tags typically consecutively placed from a sequentially numbered, commercially sourced package either at birth, spring processing, or shipping and, therefore, share the initial numeric sequence unique to a herd. The mean weight of calves from the 2020 study population was 253 kg (range 211–291 kg). Lighter-weight calves were targeted for 2021 and, hence, the mean weight was 225 kg (range 351–694 kg). On the day of purchase, calves were transported 51 km to a research feedlot at the Livestock and Forage Centre of Excellence (LFCE) in Clavet, Saskatchewan. Calves were rested in a holding pen overnight and processed the following morning.

### 4.3. Calf Processing Procedure

All animals (*n* = 1600) were processed at 1DOF following industry protocol that included the placement of a feedlot identification ear tag, verification of castration, and subcutaneous administration of *M*. *haemolytica* and a modified live viral vaccine (Pyramid^®^ 5 + Presponse^®^, Boehringer Ingelheim Animal Health, Duluth, GA, USA) and a multivalent clostridial vaccine (Ultrachoice^®^ 7, Zoetis Inc., Florham Park, NJ, USA). All calves received a growth implant (Ralgro^®^, Merck Animal Health, Rahway, NJ, USA) and a topical anthelmintic (Solmectin^TM^, Solvet, Calgary, AB, Canada). In 2020, calves (*n* = 800) received metaphylactic tulathromycin as a single dose of 2.5 mg/kg of body weight (Draxxin^®^, Zoetis Inc., Florham Park, NJ, USA), administered subcutaneously based on the average weight of the cohort. In the fall of 2021, cattle in four pens (*n* = 400 calves) were administered metaphylactic tulathromycin and cattle in the other four pens (*n* = 400 calves) were administered oxytetracycline (Oxyvet^®^200 LA, Vetoquinol, Lavaltrie, QC, Canada) subcutaneously as a single dose of 20mg/kg of body weight. Following processing, each cohort of 100 calves was placed in their designated home pen, where they remained for the duration of the study.

### 4.4. Animal Housing and Management

Calves were housed in eight outdoor, dirt floor pens, designed as per the Canadian guidelines for feedlot cattle [71]. Each pen held 100 animals and pens were filled consecutively. The four adjacent pairs of pens each shared fence-line watering bowls. The first four and last four pens had contact through consecutive cross-fences. A building separated the first four pens from the last four pens.

On day 1, calves were fed a high-forage starter feed ration (34% barley silage, 15% barley, 44% hay, 7% canola meal) to encourage bunk eating. For the remainder of the feeding period, the diet consisted of 59% barley silage, 15% barley, 20% hay, and 6% canola meal. The calves were started at an estimated 15 lbs/head on an as-fed basis (10 lbs dry-matter intake (DMI)), which was steadily increased until the calves reached 30–33 lbs as-fed or 20–22 lbs of DMI. A supplement was provided consisting of salt (1500 mg/kg) and vitamins A, D, and E. Monensin (33mg/kg dry matter, concentrate) was included in this supplement and was the only in-feed antimicrobial administered.

### 4.5. Sampling Procedures

All calves were sampled at two time points: at the time of arrival and processing (1DOF) prior to metaphylaxis administration and again at 13DOF. A random subset of calves from each pen was sampled at 36DOF as determined by available resources and laboratory capacity: 10 calves/pen in 2020 and 30 calves/pen in 2021. A snowstorm in 2021 delayed the initial sampling of one pen (#16) by a day. Thus, sampling time points occurred on 12DOF and 35DOF, respectively. The results from these samplings were incorporated into the analysis along with samples from the regular 13DOF and 36DOF sampling performed for all other pens. In addition, above-average mortalities in pen 16 (the last pen filled in the year 2021) led to mass-treatment with oxytetracycline at 30DOF. As a result, 20 calves from pen 16 were sampled pre-treatment (30DOF) and post-treatment (35DOF).

At each sampling time point, calves were restrained in a hydraulic chute and sampled via three DNP swabs; a neck extender was used to stabilize the calves’ heads during sampling. A single-use paper towel was used to wipe clean the external nares, and a double-guarded culture swab (Continental Plastic Corp., Delevan, WI, USA) was directed into the ventral meatus of the nostril. The polyester-tipped swab was advanced through the inner sheath and vigorously rotated against the nasopharyngeal mucosa for 5–6 rotations. The swab was withdrawn into the inner sheath and outer guard prior to removal from the nostril, and approximately 3 cm of swab tip was cut and placed in a 15 mL vial containing 3 mL of liquid Amies transport medium. Two additional samples were obtained from alternating nostrils using the same procedure and all three DNP swabs per calf were pooled in the same vial.

### 4.6. Bacteriology

Samples were transported to the University of Saskatchewan for same-day processing. The samples were vortexed for 1 min and a 300 uL aliquot was submitted to Prairie Diagnostic Services, Inc. (Saskatoon, SK, Canada, PDS). For *M. haemolytica*, *P. multocida*, and *H. somni* cultures, a 10 uL inoculation loop of sample was cultured on Columbia agar with 5% sheep blood (BA) and a second loop was cultured on chocolate agar (CHOC); plates were incubated at 35 °C for 18 h in 5% CO_2_. Bacterial colonies were examined at 18 h and 42 h of incubation. By examining both BA and CHOC plates, one isolate exhibiting phenotypic morphologies for each bacterium of interest was selected and confirmed using MALDI-TOF MS (Bruker Daltonik, Bremen, Germany), according to manufacturer guidelines. MALDI-TOF MS Biotyper Microflex LT Compass version 1.4 software and the MSP library were used for direct testing. If visible characteristics suggested the presence of multiple isolates of interest from one sample, representative colonies of each unique colony morphology were selected for identification. Positive and negative controls were processed for each day of sample setup and for each new media lot using *Staphylococcus aureus* ATCC 29213, *Escherichia coli* ATCC 25922, and *Histophilus somni* ATCC 700025. Only MALDI-TOF identification scores of ≥2 indicating secure species-level identification were used for further analysis. A plain matrix spot was run with every MALDI run to ensure no contamination. The diagnostic laboratory used in this study also processed the respiratory samples for the national surveillance program, CIPARS, following the same methods for bacterial isolation and identification.

### 4.7. Antimicrobial Susceptibility Testing

The AST procedures were also consistent with those used in CIPARS. This included utilizing the same AST microdilution panel and adhering to identical reporting standards for distinguishing between susceptible and resistant isolates.

For AST, all isolates that showed positive MALDI-TOF MS identification were streaked from the inoculated Todd Hewitt broth onto purity plates specific to each bacteria type: BA for *M. haemolytica* and *P. multocida* and CHOC for *H. somni*. Each colony of interest from the purity plates underwent AST using a commercially available bovine serial broth microdilution panel (Thermo Fisher Scientific^TM^, Waltham, MA, USA, Bovine AST BOPO7F Plate) on the Sensititre^TM^ platform. *Escherichia coli* ATCC 25922, *Staphylococcus aureus* ATCC 29213, and *Histophilus somni* ATCC 700025 were used as positive controls. The minimum inhibitory concentrations (MICs) plate was placed and read on the BIOMIC^®^ V3 microplate reader. The MIC value was considered equal to the lowest concentration of antimicrobial that inhibited visible growth. The MIC for each antimicrobial was compared to Clinical and Laboratory Standards Institute (CLSI) breakpoints, where available [72]. Isolates with “intermediate” MICs were categorized as “susceptible”.

MIC 50 and MIC 90 were defined as the MIC values at which ≥50% or ≥90% of the isolates were inhibited [73]. Results were summarized in distribution tables by pathogen, antimicrobial, and sampling time point (1DOF, 13DOF, and 36DOF). The MIC results are provided in the Supplementary Materials S1 (Tables S1–S9) for all antimicrobials tested, regardless of whether a CLSI breakpoint was available.

### 4.8. Treatment of Calves with BRD

Experienced feedlot personnel monitored the animals daily for signs of illness. Calves exhibiting signs of respiratory disease were identified using a DART (depression, appetite, respiratory system, temperature) BRD clinical scoring system [74]. The severity of clinical signs was graded using a standardized numerical scale ranging from 0 (clinically normal) to 4 (moribund). To meet the BRD case definition and receive treatment, calves needed to have a score of 1 or 2 with a rectal temperature ≥ 40 °C or a score of 3 or 4 regardless of temperature (and with no other obvious causes of illness).

For calves that received metaphylactic tulathromycin, a PMI (waiting period before eligibility for retreatment) of 7 d was observed. Calves developing BRD after the metaphylaxis were administered florfenicol 40 mg/kg BW and flunixin 2.2 mg flunixin/kg BW (Resflor Gold^®^, Merck Animal Health, Rahway, NJ, USA,) subcutaneously. For calves that received metaphylactic oxytetracycline, a 5 d PMI was observed. The treatment regimen for those calves was tulathromycin (Draxxin^®^, Zoetis Inc., Florham, NJ, USA) administered subcutaneously at a dose of 2.5 mg/kg of body weight. Calves were returned to their home pen following treatment.

Morbidities and mortalities associated with *H. somni* in pen 16 during the year 2021 were mass-medicated, resulting in the cooperative decision between the feedlot manager, feedlot veterinarians, and the research team to mass-treat the pen cohort at 30DOF. Animals in this pen were administered oxytetracycline (Vetoquinol, Oxyvet^®^ 200 LA, Lavaltrie, QC, Canada) subcutaneously at a dose of 20 mg/kg of BW.

### 4.9. Statistical Analysis

Data were entered and managed in a spreadsheet (Microsoft Excel, version 2401, Microsoft Corporation, Redmond, Washington, DC, USA), and analyses were completed using a commercial statistical software package (Stata/IC, version 16.1, StataCorp LLC, College Station, TX, USA). Bacterial recovery was summarized at the calf level; antimicrobial susceptibility was also summarized by reporting prevalence at the calf level rather than at the recovered isolate level (i.e., the denominators for all antimicrobial susceptibility prevalence calculations were the total number of calves, not the total number of recovered isolates). Data from this study were used to inform simulation-based sample size calculations for pen-level sampling (Supplementary Materials S1).

The total number of calves from each pen was summarized for the positive recovery of *M. haemolytica*, *P. multocida*, and *H. somni*; bacterial co-isolation; and bacteria of interest classified as resistant to antimicrobials, with breakpoints established by the CLSI [72].

The recovery of *M. haemolytica*, *P. multocida*, and *H. somni* at the calf level at 1DOF and 13DOF were each compared between years 2020 and 2021 using mixed-effects logistic regression models [52]. Differences between years in the frequency of calves from which AMR pathogens were recovered were also examined when the crude prevalence of AMR was ≥5%. Pen-level clustering was accounted for as a random intercept in all models. Models for differences between years in the recovery of bacteria of interest and bacteria with AMR at 1DOF included year as a fixed effect. Differences at 13DOF also accounted for the choice of metaphylaxis. A single fixed effect accounted for both study population year and the metaphylactic antimicrobial used: year 2020 calves treated with metaphylactic tulathromycin, year 2021 calves treated with metaphylactic tulathromycin, and year 2021 calves treated with metaphylactic oxytetracycline.

Population-averaged prevalence and 95% confidence intervals (CIs) were estimated for calves with BRD pathogens of interest, as well as for calves from which pathogens with resistance patterns of interest were recovered from mixed models for each time point, as described (1DOF, 13DOF, and 36DOF) using the variance of the random pen effects estimated from the models as follows: β^PA^ = β^SS^/(1 + 0.346 σ^2^_h_)^0.5^ [52]. Similarly, population-averaged odds ratios (ORs) were determined when summarizing relative differences among groups. Intraclass correlation coefficients (ICC values) were reported to estimate the extent of the clustering of outcomes within pens (h), as followed from the variance from the random effects for pen: ρ = σ^2^_h_/(σ^2^_h_ + π^2^/3) [52].

Differences in pathogen recovery between sampling time points (1DOF, 13DOF, and 36DOF) accounting for repeated measures on individual calves within pens were examined with three-level mixed-effects logistic regression models for each combination of year and metaphylactic treatment. For each model, year, metaphylaxis, sampling time point, and an interaction term between the year/metaphylaxis and sampling time point variables were included as fixed effects. Individual calves and calves nested within pens were set as random intercepts. Post-hoc Wald tests were used to test the significance (*p* < 0.05) of the coefficients of the interaction term for year/metaphylaxis and sampling time for each model. The likelihood of pathogen recovery at 13DOF vs. 1DOF, 36DOF vs. 1DOF, and 36DOF vs. 13DOF were then compared for calves in year 2020 treated with metaphylactic tulathromycin, calves in year 2021 treated with metaphylactic tulathromycin, and calves in year 2021 treated with metaphylactic oxytetracycline.

Similar models were repeated for AMR patterns where the crude prevalence was ≥5% for at least one sampling time to estimate differences in recovery for each antimicrobial-resistant pathogen of interest between sampling points (1DOF, 13DOF, and 36DOF). When mixed-effects models failed to converge due to sampling time points with few or zero calves, exact logistic regression models (SAS for Windows, version 9.4, Cary, NC, USA) were used to generate estimates.

Simulation models were also developed to examine the effectiveness of different sample sizes to support pen-level testing (Supplementary Materials S2, Figure S1).

## 5. Conclusions

This study highlights substantial variability in the prevalence of target BRD bacteria and antimicrobial susceptibility profiles among pens of fall-placed calves at higher risk of BRD, observed both at feedlot arrival and again at 13DOF. The culture and AST demonstrated considerable pen-level variability over time within individual cohorts of fall-placed high-risk calves as well as between years and across different metaphylaxis protocols. 

These findings emphasize the importance of pen-level management and the challenge of making antimicrobial drug choices without laboratory guidance. Sampling a subset of 20–30 calves in a feedlot pen, either near arrival in pens where cattle did not receive metaphylaxis or after a PMI in treated cattle, would allow feedlot veterinarians and managers to make more informed AMU decisions based on the risk assessment of individual pens. The use of laboratory-based results to target antimicrobial drug selection in feedlot pens will allow the industry to remain aligned with WHO recommendations [7] and establish practical antimicrobial stewardship recommendations. These are important first steps toward improving prudent AMU in feedlots. However, considering the demonstrated dynamics of bacterial populations, exploring technologies that improve turn-around times from sampling to results and provide comprehensive data on both organisms of interest and AMR are warranted. Such advancements could better support timely antimicrobial decision-making in commercial settings.

## Figures and Tables

**Table 1:** Descriptive summary of calves at time of processing including unique herd of origin (using first 12 digits of RFID tag), calf weight (kg), and standard deviation (standard dev.).

Year	Pen	Unique Herds of Origin	Avg. Weight (kg)	Standard Dev. (kg)
2020	1	34	257	13
	2	81	243	10
	3	48	254	11
	4	43	253	13
	5	31	260	9
	6	38	262	11
	7	41	256	13
	8	30	239	13
	Combined	292	253	14
2021	9	31	223	18
	10	36	229	14
	11	33	222	14
	12	35	229	10
	13	23	220	18
	14	12	223	20
	15	38	228	13
	16	38	230	12
	Combined	208	225	15

**Table 2:** Percentage of calves from each pen cohort from which *M. haemolytica*, *P. multocida*, and *H. somni* were recovered at 1DOF (*n* = 799 calves) at on-arrival processing and before metaphylaxis, 13DOF (*n* = 798), and 36DOF (*n* = 80) for the 2020 study population.

Metaphylaxis Drug	Pen	Sampling Time	No. Calves	Recovery Rates of BRD Pathogens from Calves		
				*M. haemolytica*	*P. multocida*	*H. somni*
tulathromycin	1	1DOF	100	26%	32%	4%
		13DOF	100	77%	2%	0%
		36DOF	10	80%	20%	30%
tulathromycin	2	1DOF	100	35%	47%	3%
		13DOF	100	84%	7%	1%
		36DOF	10	70%	10%	50%
tulathromycin	3	1DOF	100	47%	48%	6%
		13DOF	100	70%	13%	0%
		36DOF	10	80%	40%	30%
tulathromycin	4	1DOF	100	47%	55%	4%
		13DOF	100	80%	21%	4%
		36DOF	10	80%	20%	60%
tulathromycin	5	1DOF	100	33%	67%	12%
		13DOF	100	14%	11%	2%
		36DOF	10	80%	10%	40%
tulathromycin	6	1DOF	99	33%	63%	7%
		13DOF	99	9%	8%	5%
		36DOF	10	70%	20%	30%
tulathromycin	7	1DOF	100	28%	70%	12%
		13DOF	99	30%	11%	12%
		36DOF	10	70%	30%	100%
tulathromycin	8	1DOF	100	15%	70%	29%
		13DOF	100	57%	8%	11%
		36DOF	10	70%	20%	50%

**Table 3:** Percentage of calves from each pen cohort from which *M. haemolytica*, *P. multocida*, and *H. somni* were recovered at 1DOF (*n* = 800 calves) at on-arrival processing and before metaphylaxis, 13DOF (*n* = 798 calves), and 36DOF (*n* = 230 calves) for the 2021 study population.

Metaphylaxis Drug	Pen	Sampling Time	No. Calves	Recovery Rates of BRD Pathogens from Calves		
				*M. haemolytica*	*P. multocida*	*H. somni*
oxytetracycline	9	1DOF	100	60%	23%	6%
		13DOF	100	61%	35%	10%
		36DOF	30	33%	33%	70%
oxytetracycline	10	1DOF	100	49%	29%	0%
		13DOF	100	52%	60%	5%
		36DOF	30	13%	57%	73%
oxytetracycline	11	1DOF	100	40%	35%	4%
		13DOF	100	47%	18%	19%
		37DOF	30	47%	20%	70%
tulathromycin	12	1DOF	100	58%	39%	7%
		13DOF	100	25%	7%	4%
		36DOF	30	63%	13%	70%
tulathromycin	13	1DOF	100	47%	29%	13%
		13DOF	100	17%	14%	21%
		36DOF	30	67%	27%	53%
tulathromycin	14	1DOF	99	48%	51%	8%
		13DOF	99	33%	14%	6%
		36DOF	30	50%	23%	73%
tulathromycin	15	1DOF	100	43%	56%	9%
		13DOF	99	44%	12%	9%
		36DOF	30	43%	20%	40%
oxytetracycline	16	1DOF	100	43%	73%	7%
		12DOF	99	61%	41%	13%
		30DOF	20	20%	35%	60%

**Table 4:** Differences in recovery of *M. haemolytica*, *P. multocida*, and *H. somni* at arrival to feedlot and prior to metaphylaxis administration (1DOF) between year 2021 and year 2020, reported as population-averaged odds ratios (ORs) and 95% confidence intervals (CIs), accounting for clustering at the pen level. *n* = 1599 calves.

Bacteria	OR 2021 vs. 2020	95% CI	*p*-Value
*M. haemolytica*	1.9	1.3, 2.8	<0.001
*P. multocida*	0.6	0.3, 0.97	0.04
*H. somni*	0.7	0.4, 1.5	0.42

**Table 5:** Differences in recovery of *M. haemolytica*, *P. multocida*, and *H. somni* at 13DOF between years and metaphylaxis administered (year 2020/tulathromycin, year 2021/tulathromycin, and year 2021/oxytetracycline), reported as population-averaged odds ratios (ORs) and 95% confidence intervals (CIs), accounting for clustering at the pen level. *n* = 1596 calves.

Bacteria	Year/Metaphylaxis Comparison	OR	95% CI	*p*-Value
*M. haemolytica*	2021/tulathromycin vs. 2020/tulathromycin	0.4	0.1, 1.3	0.12
	2021/oxytetracycline vs. 2020/tulathromycin	1.2	0.3, 4.0	0.82
	2021/oxytetracycline vs. 2021/tulathromycin	3.1	0.7, 13	0.12
*P. multocida*	2021/tulathromycin vs. 2020/tulathromycin	1.2	0.6, 2.4	0.55
	2021/oxytetracycline vs. 2020/tulathromycin	5.5	2.9, 9.3	<0.001
	2021/oxytetracycline vs. 2021/tulathromycin	4.4	2.1, 9.3	<0.001
*H. somni*	2021/tulathromycin vs. 2020/tulathromycin	2.6	0.6, 2.4	0.06
	2021/oxytetracycline vs. 2020/tulathromycin	3.3	1.2, 3.7	0.018
	2021/oxytetracycline vs. 2021/tulathromycin	1.3	0.4, 3.7	0.67

**Table 6:** Pairwise comparisons from the repeated measures and multilevel logistic regression models for the likelihood of bacterial recovery (*M. haemolytica*, *P. multocida*, *H. somni*) from calves within pens at 1DOF at on-arrival processing and before metaphylaxis, 13DOF, and 36DOF, stratified by sampling year and metaphylactic antimicrobial administered. Differences reported population-averaged odds ratios (ORs) and 95% confidence intervals (CIs) conditioned on pen and calf levels. *n* = 1599 calves at 1DOF, 1596 at 13DOF, and 310 at 36DOF.

Bacteria	Year/Metaphylaxis	DOF Comparison	OR	95% CI	*p*-Value
*M. haemolytica*	2020/tulathromycin	13DOF vs. 1DOF	2.4	1.9, 2.9	<0.001
		36DOF vs. 1DOF	6.8	4.0, 12	<0.001
		36DOF vs. 13DOF	2.8	1.7, 4.8	<0.001
	2021/tulathromycin	13DOF vs. 1DOF	0.5	0.3, 0.6	<0.001
		36DOF vs. 1DOF	1.3	0.9, 1.9	0.19
		36DOF vs. 13DOF	2.9	1.9, 4.3	<0.001
	2021/oxytetracycline	13DOF vs. 1DOF	1.3	1.0, 1.7	0.041
		36DOF vs. 1DOF	0.5	0.3, 0.7	<0.001
		36DOF vs. 13DOF	0.3	0.2, 0.5	<0.001
*P. multocida*	2020/tulathromycin	13DOF vs. 1DOF	0.1	0.1, 0.1	<0.001
		36DOF vs. 1DOF	0.2	0.1, 0.3	<0.001
		36DOF vs. 13DOF	2.3	1.3, 4	0.004
	2021/tulathromycin	13DOF vs. 1DOF	0.2	0.1, 0.2	<0.001
		36DOF vs. 1DOF	0.3	0.2, 0.5	<0.001
		36DOF vs. 13DOF	1.9	1.2, 3.2	0.01
	2021/oxytetracycline	13DOF vs. 1DOF	0.9	0.7, 1.2	0.65
		36DOF vs. 1DOF	0.9	0.6, 1.3	0.55
		36DOF vs. 13DOF	0.9	0.6, 1.4	0.75
*H. somni*	2020/tulathromycin	13DOF vs. 1DOF	0.5	0.3, 0.7	<0.001
		36DOF vs. 1DOF	9.8	5.7, 17	<0.001
		36DOF vs. 13DOF	22	12, 40	<0.001
	2021/tulathromycin	13DOF vs. 1DOF	1.1	0.7, 1.7	0.70
		36DOF vs. 1DOF	13	7.7, 22	<0.001
		36DOF vs. 13DOF	12	7.1, 20	<0.001
	2021/oxytetracycline	13DOF vs. 1DOF	2.7	1.6, 4.6	<0.001
		36DOF vs. 1DOF	44	23, 84	<0.001
		36DOF vs. 13DOF	16	9.3, 27	<0.001

**Table 7:** Number of calves with bacterial co-isolation patterns recovered at 1DOF at on-arrival processing and before metaphylaxis and 13DOF stratified by sampling year and metaphylactic antimicrobial administered.

				Number (%) of Calves with Bacterial Co-Isolation Pattern							
Year	Meta. ^1^	Time Point	No. Calves	Neg. Culture	MH	PM	HS	MH + PM	MH + HS	PM + HS	MH + PM + HS
2020	Tula	1DOF	799	183 (23%)	133 (17%)	286 (36%)	26 (3%)	120 (15%)	6 (0.8%)	40 (5%)	5 (0.6%)
		13DOF	798	322 (40%)	366 (46%)	32 (4%)	19 (2%)	43 (5%)	10 (1%)	4 (0.5%)	2 (0.3%)
2021	Tula	1DOF	400	83 (21%)	117 (29%)	94 (24%)	19 (5%)	69 (17%)	7 (12%)	8 (2%)	3 (0.8%)
		13DOF	399	219 (55%)	102 (26%)	26 (6.5%)	27 (7%)	12 (3%)	4 (1%)	8 (2%)	1 (0.3%)
2021	Oxy	1DOF	400	102 (26%)	131 (33%)	96 (24%)	5 (1%)	54 (14%)	2 (0.5%)	5 (1%)	5 (1%)
		13DOF	399	84 (21%)	133 (33%)	71 (18%)	13 (3%)	64 (16%)	15 (4%)	11 (3%)	8 (2%)
All Years	All Groups	1DOF	1599	368 (23%)	381 (24%)	476 (30%)	50 (3%)	243 (15%)	15 (0.9%)	53 (3%)	13 (0.8%)
		13DOF	1596	625 (39%)	601 (38%)	129 (8%)	59 (3%)	119 (8%)	29 (2%)	23 (1%)	11 (0.7%)

^1^ Antimicrobial used for metaphylaxis (Meta.): Tula, tulathromycin; Oxy, oxytetracycline; MH, *M. haemolytica*; PM, *P. multocida*; HS, *H. somni*.

**Table 8:** Number (%) of calves with bacteria interpreted as resistant to select antimicrobials ^1^ at 1DOF at on-arrival processing and before metaphylaxis, 13DOF, and 36DOF for the 2020 study population based on CLSI minimum inhibitory concentration breakpoints (all calves received metaphylactic tulathromycin at arrival).

Time Point	No. Calves	Bacteria ^2^	Number (%) of Calves with Isolates Resistant to Select Antimicrobials ^1^								
			AMP	DANO	FLOR	SPECT	TET	GAM	TILD	TILM	TUL
1DOF	799	MH	1 (0.1%)	0	0	0	0	1 (0.1%)	0	0	1 (0.1%)
	799	PM	15 (2%)	0	0	31 (4%)	28 (4%)	0	0	NI	0
	799	HS	0	NI	0	1 (0.1%)	0	0	4 (0.5%)	NI	6 (0.8%)
13DOF	798	MH	2 (0.3%)	0	1 (0.1%)	0	1 (0.1%)	351 (44%)	3 (0.4%)	25 (3%)	341 (43%)
	798	PM	7 (0.9%)	0	0	10 (1%)	8 (1%)	1 (0.1%)	1 (0.1%)	NI	1 (0.1%)
	798	HS	0	NI	0	0	0	1 (0.1%)	2 (0.3%)	NI	2 (0.3%)
36DOF	80	MH	2 (2.5%)	0	0	0	1 (1.3%)	44 (55%)	1 (1.3%)	7 (8.8%)	40 (50%)
	80	PM	0	0	0	3 (3.8%)	3 (3.8%)	0	0	NI	0
	80	HS	0	NI	0	0	0	0	0	NI	0

^1^ Antimicrobials for which isolates were tested: AMP, ampicillin; DANO, danofloxacin; FLOR, florfenicol; SPECT, spectinomycin; TET, tetracycline; GAM, gamithromycin; TILD, tildipirosin; TILM, tilmicosin; TUL, tulathromycin. No resistance observed for penicillin, ceftiofur, or enrofloxacin. ^2^ MH, *M. haemolytica*; PM, *P. multocida*; HS, *H. somni.* NI = not interpretable, CLSI breakpoints not available.

**Table 9:** Number (%) of calves with bacteria interpreted as resistant to select antimicrobials ^1^ at 1DOF at on-arrival processing and before metaphylaxis, 13DOF, and 36DOF for the 2021 study population based on CLSI minimum inhibitory concentration breakpoints, stratified by metaphylactic antimicrobial administered at arrival.

				Number (%) of Calves with Isolates Resistant to Select Antimicrobials ^1^								
Meta. ^2^	Time Point	No. Calves	Bacteria ^3^	AMP	PEN	DANO	SPECT	TET	GAM	TILD	TILM	TUL
Tula	1DOF	399	MH	2 (0.5%)	0	2 (0.5%)	0	1 (0.3%)	0	1 (0.3%)	2 (0.5%)	0
		399	PM	9 (2%)	0	0	5 (1%)	3 (0.8%)	0	0	NI	0
		399	HS	0	0	NI	1 (0.3%)	0	0	0	NI	0
	13DOF	399	MH	0	0	0	0	18 (5%)	27 (7%)	38 (10%)	43 (11%)	27 (7%)
		399	PM	6 (2%)	0	0	0	0	0	0	NI	0
		399	HS	1 (0.3%)	0	NI	1 (0.3%)	1 (0.3%)	0	0	NI	0
	36DOF	119	MH	1 (0.8%)	0	0	0	23 (19.3%)	10 (8.4%)	12 (10.1%)	12 (10.1%)	10 (8.4%)
		119	PM	0	0	0	0	0	0	0	NI	0
		119	HS	3 (2.5%)	1 (0.8%)	0	0	6 (5.0%)	0	0	NI	0
Oxy	1DOF	399	MH	0	1 (0.3%)	0	0	1 (0.3%)	0	0	0	0
		399	PM	8 (2%)	0	0	11 (3%)	9 (2%)	0	0	NI	0
		399	HS	0	0	NI	0	1 (0.3%)	0	0	NI	0
	13DOF	398	MH	1 (0.3%)	2 (0.5%)	0 (0%)	0	3 (0.8%)	3 (0.8%)	3 (0.8%)	3 (0.8%)	3 (0.8%)
		398	PM	3 (0.8%)	0	0 (0%)	53 (13%)	52 (13%)	0	0	NI	0
		398	HS	0	0	NI	0	14 (4%)	0	0	NI	0
	36DOF	110	MH	0	0	0	0	0	0	0	0	0
		110	PM	2 (1.8%)	1 (0.9%)	0	15 (13.6%)	17 (15.5%)	0	0	NI	0
		110	HS	1 (0.9%)	0	NI	0	11 (10%)	0	0	NI	0

^1^ Antimicrobials for which isolates were tested: AMP, ampicillin; PEN, penicillin; DANO, danofloxacin; SPECT, spectinomycin; TET, tetracycline; GAM, gamithromycin; TILD, tildipirosin; TILM, tilmicosin; TUL, tulathromycin. No resistance observed for ceftiofur, florfenicol, or enrofloxacin. ^2^ Antimicrobial used for metaphylaxis (Meta.). ^3^ MH, *M. haemolytica*; PM, *P. multocida*; HS, *H. somni*. NI = not interpretable, CLSI breakpoints not available.

**Table 10:** Differences in antimicrobial resistance (AMR) patterns from the repeated measures multilevel logistic regression models at 13DOF between years and metaphylaxis administered (year 2020/tulathromycin, year 2021/tulathromycin, and year 2021/oxytetracycline), reported as population-averaged odds ratios (ORs) and 95% confidence intervals (CIs), accounting for clustering at the pen-level. *n* = 1595 calves for which susceptibility data were available.

AMR Outcome of Interest	Pairwise Comparison of Year/Metaphylaxis	OR	95% CI	*p*-Value
*M. haemolytica* tulathromycin	2021/tulathromycin vs. 2020/tulathromycin	0.1	0.03, 0.7	0.02
	2021/oxytetracycline vs. 2020/tulathromycin	0.06	0.01, 0.4	0.004
	2021/oxytetracycline vs. 2021/tulathromycin	0.4	0.05, 3.2	0.39
*M. haemolytica* gamithromycin	2021/tulathromycin vs. 2020/tulathromycin	0.1	0.03, 0.7	0.018
	2021/oxytetracycline vs. 2020/tulathromycin	0.06	0.01, 0.4	0.004
	2021/oxytetracycline vs. 2021/tulathromycin	0.4	0.05, 3.1	0.39
*P. multocida* tetracycline *	2021/tulathromycin vs. 2020/tulathromycin **	0.2	0, 0.90	0.08
	2021/oxytetracycline vs. 2020/tulathromycin	15	6.9, 37	<0.0001
	2021/oxytetracycline vs. 2021/tulathromycin **	83	19, ∞	<0.0001

Post-hoc Wald test for significance of differences between year and metaphylaxis options was not different for the recovery of any pathogen with AMR or *M. haemolytica* with resistance to tildipirosin, tilmicosin, or tetracycline. * Zero calves with tetracycline resistance at 13DOF from year 2021 tulathromycin-treated pens. Exact logistic regression used. ** Median unbiased estimate reported.

**Table 11:** Pairwise comparisons from the repeated measures, multilevel logistic regression models for the likelihood of a calf within a pen having *M. haemolytica* with antimicrobial resistance (AMR) to tulathromycin, gamithromycin, tilmicosin, tildipirosin, or tetracycline across time for each year and metaphylaxis option, reported as population-averaged odds ratios (ORs) and 95% confidence intervals (CIs), accounting for clustering at the pen level. *n* = 1595 calves for which susceptibility data were available.

AMR Outcome of Interest	Year/Metaphylaxis	DOF Comparison	OR	95% CI	*p*-Value
Tulathromycin	2020/tulathromycin	13DOF vs. 1DOF	151	36, 638	<0.001
		36DOF vs. 1DOF	206	46, 915	<0.001
		36DOF vs. 13DOF	1.4	0.9, 2.1	0.14
	2021/tulathromycin *	13DOF vs. 1DOF **	41	9.1, ∞	<0.001
		36DOF vs. 1DOF **	50	10, ∞	<0.001
		36DOF vs. 13DOF	1.3	0.5, 3.4	0.67
	2021/oxytetracycline	13DOF vs. 1DOF **	3.9	0.6, ∞	0.25
		36DOF vs. 1DOF	·	·	·
		36DOF vs. 13DOF **	0.9	0, 6.2	0.96
Gamithromycin	2020/tulathromycin	13DOF vs. 1DOF	160	38, 672	<0.001
		36DOF vs. 1DOF	260	58, 1151	<0.001
		36DOF vs. 13DOF	1.6	1.1, 2.5	0.02
	2021/tulathromycin *	13DOF vs. 1DOF **	41	9.1, ∞	<0.001
		36DOF vs. 1DOF **	50	10, ∞	<0.001
		36DOF vs. 13DOF	1.3	0.5, 3.4	0.66
	2021/oxytetracyclin	13DOF vs. 1DOF **	3.9	0.6, ∞	0.25
		36DOF vs. 1DOF	·	·	·
		36DOF vs. 13DOF **	0.90	0, 6.2	0.96
Tildipirosin	2020/tulathromycin *	13DOF vs. 1DOF **	3.9	0.6, ∞	0.25
		36DOF vs. 1DOF **	9.9	0.5, ∞	0.18
		36DOF vs. 13DOF	3.3	0.06, 42	0.64
	2021/tulathromycin	13DOF vs. 1DOF	10	3.0, 34	<0.001
		36DOF vs. 1DOF	11	3.0, 37	<0.001
		36DOF vs. 13DOF	1.0	0.7, 1.6	0.85
	2021/oxytetracycline *	13DOF vs. 1DOF **	3.9	0.6, ∞	0.12
		36DOF vs. 1DOF	·	·	·
		36DOF vs. 13DOF **	0.9	0, 6.2	0.48
Tilmicosin	2020/tulathromycin *	13DOF vs. 1DOF **	37	8.1, ∞	<0.001
		36DOF vs. 1DOF **	103	20, ∞	<0.001
		36DOF vs. 13DOF	3.0	1.0, 7.4	0.04
	2021/tulathromycin	13DOF vs. 1DOF	6.6	2.9, 15	<0.001
		36DOF vs. 1DOF	6.4	2.6, 16	<0.001
		36DOF vs. 13DOF	1.0	0.6, 1.5	0.84
	2021/oxytetracycline *	13DOF vs. 1DOF **	3.9	0.6, ∞	0.25
		36DOF vs. 1DOF	·	·	·
		36DOF vs. 13DOF **	0.9	0, 6.2	0.96
Tetracycline	2020/tulathromycin *	13DOF vs. 1DOF **	1.0	0.05, ∞	1.00
		36DOF vs. 1DOF **	10	0.5, ∞	0.18
		36DOF vs. 13DOF	10	0.1, 1000	0.35
	2021/tulathromycin	13DOF vs. 1DOF	7.2	1.9, 27	0.001
		36DOF vs. 1DOF	27	6.3, 115	<0.001
		36DOF vs. 13DOF	3.7	2, 7	<0.001
	2021/oxytetracycline *	13DOF vs. 1DOF	3.0	0.2, 159	0.62
		36DOF vs. 1DOF **	3.6	0, 69	1.57
		36DOF vs. 13DOF **	0.9	0, 6.2	0.96

* Exact logistic regression equation performed. · odds ratio (OR) was not estimable. ** Indicates a median unbiased estimate.

**Table 12:** Pairwise comparisons from the repeated measures, multilevel logistic regression models for the likelihood of a calf within a pen having *P. multocida* with tetracycline resistance or spectinomycin resistance (antimicrobial resistance (AMR)) across time points for each year and metaphylaxis option, reported as population-averaged odds ratios (ORs) and 95% confidence intervals (CIs), accounting for clustering at the pen level. *n* = 1595 calves for which susceptibility data were available.

AMR Outcome of Interest	Year/Metaphylaxis	DOF Comparison	OR	95% CI	*p*-Value
Tetracycline	2020/tulathromycin	13DOF vs. 1DOF	0.4	0.2, 0.7	0.001
		36DOF vs. 1DOF	1.1	0.4, 2.7	0.83
		36DOF vs. 13DOF	2.9	1, 8.2	0.052
	2021/tulathromycin *	13DOF vs. 1DOF **	0.3	0, 1.7	0.25
		36DOF vs. 1DOF **	0.9	0, 5.8	0.91
		36DOF vs. 13DOF	·	·	·
	2021/oxytetracycline	13DOF vs. 1DOF	5.1	2.8, 9.3	≤0.001
		36DOF vs. 1DOF	6.0	2.9, 12.6	≤0.001
		36DOF vs. 13DOF	1.2	0.7, 1.9	0.50
Spectinomycin	2020/tulathromycin	13DOF vs. 1DOF	0.41	0.2, 0.7	0.001
		36DOF vs. 1DOF	1.02	0.4, 2.6	0.97
		36DOF vs. 13DOF	2.5	0.9, 6.9	0.084
	2021/tulathromycin *	13DOF vs. 1DOF **	0.1	0, 0.8	0.06
		36DOF vs. 1DOF **	0.5	0, 2.8	0.54
		36DOF vs. 13DOF	·	·	·
	2021/oxytetracycline	13DOF vs. 1DOF	4.3	2.5, 7.5	≤0.001
		36DOF vs. 1DOF	4.3	2.2, 8.3	≤0.001
		36DOF vs. 13DOF	1.0	0.6, 1.6	0.97

* Exact logistic regression equation performed. · odds ratio (OR) was not estimable. ** Indicates a median unbiased estimate.

## Data Availability

The original contributions presented in this study are included in the article/Supplementary Material; further inquiries can be directed to the corresponding author.

## Supplementary Materials

The following supporting information can be downloaded at https://www.mdpi.com/article/10.3390/antibiotics13040322/s1: Supplementary Materials S1—Tables S1–S9: Supplemental MIC tables. Supplementary Materials S2—Figure S1: Simulation model to evaluate sample size estimates in support of pen-level testing.
