# Supplementary material for: Variation in Pen-Level Prevalence of BRD Bacterial Pathogens and Antimicrobial Resistance Following Feedlot Arrival in Beef Calves
Source: Antibiotics (Basel). 2024 Apr 2;13(4):322. doi: 10.3390/antibiotics13040322 (PMC11047553; doi:10.3390/antibiotics13040322)
Supplement: Supplementary file 1 [file antibiotics-13-00322-s001.zip › antibiotics-2891391-supplementary.pdf]

Supplementary Materials S1- Supplemental MIC Tables

From:

Variation in Pen-Level Prevalence of BRD Bacterial Pathogens and Antimicrobial Resistance Following Feedlot Arrival in Beef Calves

Jennifer N. Abi Younes [1], John R. Campbell [1], Simon J. G. Otto [2], Sheryl P. Gow [3], Amelia R. Woolums [4], Murray Jelinski [1], Stacey Lacoste [1] and Cheryl L. Waldner [1,*]

[1] Department of Large Animal Clinical Sciences, Western College of Veterinary Medicine, University of Saskatchewan, Saskatoon, SK S7N 5B4, Canada; jennifer.abiyounes@usask.ca (J.N.A.Y.); john.campbell@usask.ca (J.R.C.); murray.jelinski@usask.ca (M.J.); srl676@mail.usask.ca (S.L.)

[2] School of Public Health, University of Alberta, Edmonton, AB T6G 1C9, Canada; simon.otto@ualberta.ca

[3] Canadian Integrated Program for Antimicrobial Resistance Surveillance, Public Health Agency of Canada, Saskatoon, SK S7L 0Z2, Canada; sheryl.gow@phac-aspc.ca

[4] Department of Pathobiology and Population Medicine, College of Veterinary Medicine, Mississippi State University, Mississippi State, MS 39762, USA; amelia.woolums@msstate.edu

* Correspondence: cheryl.waldner@usask.ca

Table S1. Distribution of minimum inhibitory concentrations among *M. haemolytica* isolates from year 2020 tulathromycin-treated calves.

Table S2. Distribution of minimum inhibitory concentrations among *P. multocida* isolates from year 2020 tulathromycin-treated calves.

Table S3. Distribution of minimum inhibitory concentrations among *H.somni* isolates from year 2020 tulathromycin-treated calves.

Table S4. Distribution of minimum inhibitory concentrations among *M. haemolytics* isolates from year 2021 tulathromycin-treated calves.

Table S5. Distribution of minimum inhibitory concentrations among *P. multocida* isolates from year 2021 tulathromycin-treated calves.

Table S6. Distribution of minimum inhibitory concentrations among *H. somni* isolates from year 2021 tulathromycin-treated calves.

Table S7. Distribution of minimum inhibitory concentrations among *M. haemolytica* isolates from year 2021 oxytetracycline-treated calves.

Table S8. Distribution of minimum inhibitory concentrations among *P. multocida* isolates from year 2021 oxytetracycline-treated calves.

Table S9. Distribution of minimum inhibitory concentrations among *H. somni* isolates from year 2021 oxytetracycline-treated calves.

Table S1. Distribution of minimum inhibitory concentrations among *M. haemolytica* isolates from year 2020 tulathromycin-treated calves.

Category[a]	Antimicrobial	Sampling time	No. Isolates	No. Calves	MIC 50	MIC 90	% R	Distribution (%) of MICs (µg/mL)														
								0.03	0.06	0.12	0.25	0.5	1	2	4	8	16	32	64	128	256	512
I	Ceftiofur	1DOF	264	799	≤0.253	≤0.25	0				99.2	0.4		0.4								
		13DOF	421	798	≤0.25	≤0.25	0				99.3	0.2	0.2		0.2							
		36DOF	60	80	≤0.25	≤0.25	0				98.3	1.7										
	Danofloxacin	1DOF	264	799	≤0.12	≤0.12	0			100												
		13DOF	421	798	≤0.12	≤0.12	0			99.8	0.2											
		36DOF	60	80	≤0.12	≤0.12	0			100.0												
	Enrofloxacin	1DOF	264	799	≤0.12	≤0.12	0			100.0												
		13DOF	421	798	≤0.12	≤0.12	0			99.8	0.2											
		36DOF	60	80	≤0.12	≤0.12	0			100												
II	Ampicillin[b]	1DOF	264	799	≤0.25	≤0.25	0.4				99.6	**0.4**										
		13DOF	421	798	≤0.25	≤0.25	0.5				99.5	**0.5**										
		36DOF	60	80	≤0.25	≤0.25	3.3				96.7	**3.3**										
	Clindamycin	1DOF	264	799	8	16	N/I								5.3	57.2	37.1	0.4				
		13DOF	421	798	8	16	N/I							0.2	17.1	68.9	13.3	0.5				
		36DOF	60	80	8	16	N/I							1.7	8.3	58.3	31.7					
	Gamithromycin	1DOF	264	799	≤ 1	≤ 1	0.4						90.5	8.7		0.4	**0.4**					
		13DOF	421	798	> 8	> 8	83.4						11.2	2.1	3.1	0.2	**83.4**					
		36DOF	60	80	> 8	> 8	73.3						25.0	1.7			**73.3**					
	Gentamicin	1DOF	264	799	2	2	N/I						14.8	83.7	1.5							
		13DOF	421	798	2	2	N/I						27.3	64.9	7.8							
		36DOF	60	80	2	4	N/I						18.3	68.3	13.3							
	Neomycin	1DOF	264	799	4	8	N/I								72.0	20.8	0.8	2.3	4.2			
		13DOF	421	798	4	16	N/I								62.5	26.6	4.3	1.7	5.0			
		36DOF	60	80	4	> 32	N/I								66.7	18.3	1.7		13.3			
	Penicillin	1DOF	264	799	≤0.12	≤0.12	0			90.9	8.3	0.8										
		13DOF	421	798	≤0.12	0.25	0			89.1	8.6	2.4										

								Distribution (%) of MICs (μg/mL)														
**Category[a]**	**Antimicrobial**	**Sampling time**	**No. Isolates**	**No. Calves**	**MIC 50**	**MIC 90**	**% R**	**0.03**	**0.06**	**0.12**	**0.25**	**0.5**	**1**	**2**	**4**	**8**	**16**	**32**	**64**	**128**	**256**	**512**
		36DOF	60	80	≤0.12	0.25	0			86.7	11.7	1.7										
	Spectinomycin	1DOF	264	799	32	32	0										2.3	97.7				
		13DOF	421	798	32	32	0										1.4	98.6				
		36DOF	60	80	32	32	0										3.3	96.7				
	Tiamulin	1DOF	264	799	16	16	N/I								0.8	8.3	84.9	6.1				
		13DOF	421	798	16	16	N/I									7.8	89.3	2.6	0.2			
		36DOF	60	80	16	16	N/I									5.0	93.3	1.7				
	Tildipirosin	1DOF	264	799	≤1	2	0						84.1	9.5	0.8	5.7						
		13DOF	421	798	≤1	2	0.7						57.7	34.0	1.4	6.2	**0.7**					
		36DOF	60	80	≤1	8	1.7						73.3	13.3		11.7		**1.7**				
	Tilmicosin	1DOF	264	799	4	8	0.0							3.4	51.1	39.8	5.7					
		13DOF	421	798	16	16	5.9							1.2	6.9	29.0	57.0	**5.9**				
		36DOF	60	80	16	>16	11.7							1.7	21.7	20.0	45.0	**11.7**				
	Trimethoprim-sulfamethoxazole	1DOF	264	799	≤2	≤2	N/I							99.2	0.8							
		13DOF	421	798	≤2	≤2	N/I							99.8	0.2							
		36DOF	60	80	≤2	≤2	N/I							98.3	1.7							
	Tulathromycin	1DOF	264	799	≤8	≤8	0.4									93.6	6.1		**0.4**			
		13DOF	421	798	64	>64	81.0									11.9	0.7	6.4	**68.7**	**12.4**		
		36DOF	60	80	64	64	66.7									25.0	1.7	6.7	**65.0**	**1.7**		
	Tylosin	1DOF	264	799	>32	>32	N/I											2.7	97.4			
		13DOF	421	798	>32	>32	N/I											0.7	99.3			
		36DOF	60	80	>32	>32	N/I												100.0			
**III**	Florfenicol	1DOF	264	799	0.5	1	0					57.6	42.4									
		13DOF	421	798	0.5	1	0.2				0.7	77.4	21.4	0.2		**0.2**						
		36DOF	60	80	0.5	1	0					71.7	28.3									

								Distribution (%) of MICs (μg/mL)														
Category[a]	Antimicrobial	Sampling time	No. Isolates	No. Calves	MIC 50	MIC 90	% R	0.03	0.06	0.12	0.25	0.5	1	2	4	8	16	32	64	128	256	512
	Sulphadimethoxine	1DOF	264	799	≤256	≤256	N/I														91.7	8.3
		13DOF	421	798	>256	>256	N/I														12.4	87.7
		36DOF	60	80	>256	>256	N/I														21.7	78.3
	Tetracycline	1DOF	264	799	≤0.5	1	0					87.5	5.7	1.1	5.7							
		13DOF	421	798	≤0.5	≤0.5	0.2					91.0	2.4	0.7	5.7		**0.2**					
		36DOF	60	80	≤0.5	4	1.7					85.0	1.7		11.7		**1.7**					

Pathogen MIC breakpoints were based on references available by the Clinical and Laboratory Standards Institute (CLSI) [CLSI VET01S (2023)].

Based on CLSI breakpoints, susceptible isolates are indicated by a white background, intermediate by a medium grey background, and resistant by a dark grey background.

The thick vertical black line indicates the CLSI established resistant breakpoint MIC when available.

The black-outlined cells represent the MIC dilution ranges that were tested. If growth was observed at the highest antimicrobial tested, the MIC was assigned to the next dilution and will appear outside the outlined area, while those with MIC concentrations one dilution lower than the tested range will be situated below it.

[a] Categorization of antimicrobial based on importance in human medicine as defined by the Veterinary Drug Directorate.

[b] For ampicillin, the current breakpoint for susceptible isolates is ≤0.03, 0.06-0.12 for intermediate, and ≥0.25 μg/ml for resistant. However, the lowest concentration available on the sensitivity panel is ≤0.25.

Table S2. Distribution of minimum inhibitory concentrations among *P. multocida* isolates from year 2020 tulathromycin-treated calves.

								Distribution (%) of MICs (μg/mL)														
Category[a]	Antimicrobial	Sampling time	No. Isolates	No. Calves	MIC 50	MIC 90	% R	0.03	0.06	0.12	0.25	0.5	1	2	4	8	16	32	64	128	256	512
I	Ceftiofur	1DOF	451	799	≤0.25	≤0.25	0				99.8	0.2										
		13DOF	81	798	≤0.25	≤0.25	0				100											
		36DOF	17	80	≤0.25	≤0.25	0				100											
	Danofloxacin	1DOF	451	799	≤0.12	≤0.12	0			100												
		13DOF	81	798	≤0.12	≤0.12	0			100												
		36DOF	17	80	≤0.12	≤0.12	0			100												
	Enrofloxacin	1DOF	451	799	≤0.12	≤0.12	0			99.8	0.2											
		13DOF	81	798	≤0.12	≤0.12	0			100												
		36DOF	17	80	≤0.12	≤0.12	0			100												
II	Ampicillin[b]	1DOF	451	799	≤0.25	≤0.25	3.3				96.7	**3.3**										
		13DOF	81	798	≤0.25	≤0.25	8.6				91.4	**8.6**										
		36DOF	17	80	≤0.25	≤0.25	0				100											
	Clindamycin	1DOF	451	799	>16	>16	N/I								0.2	0.7	6.7	92.5				
		13DOF	81	798	>16	>16	N/I										4.9	95.1				
		36DOF	17	80	>16	>16	N/I								5.9		11.8	82.4				
	Gamithromycin	1DOF	451	799	≤1	≤1	0						99.1	0.9								
		13DOF	81	798	≤1	≤1	1.2						91.4	4.9	2.5		**1.2**					
		36DOF	17	80	≤1	2	0						88.2	5.9		5.9						
	Gentamicin	1DOF	451	799	4	8	N/I						0.9	19.7	57.2	22.0	0.2					
		13DOF	81	798	4	8	N/I						4.9	11.1	54.3	29.6						
		36DOF	17	80	4	8	N/I							35.3	47.1	17.7						
	Neomycin	1DOF	451	799	16	32	N/I								14.2	14.6	57.9	3.8	9.5			
		13DOF	81	798	16	>32	N/I								7.4	8.6	55.6	12.4	16.1			
		36DOF	17	80	16	>32	N/I								23.5	11.8	35.3	5.9	23.5			

Category[a]	Antimicrobial	Sampling time	No. Isolates	No. Calves	MIC 50	MIC 90	% R	Distribution (%) of MICs (µg/mL)														
								0.03	0.06	0.12	0.25	0.5	1	2	4	8	16	32	64	128	256	512
	Penicillin	1DOF	451	799	≤0.12	0.25	0			75.4	24.6											
		13DOF	81	798	≤0.12	0.25	0			79.0	21.0											
		36DOF	17	80	≤0.12	0.25	0			82.4	11.8	5.9										
	Spectinomycin	1DOF	451	799	32	32	6.9									0.4	37.7	54.8	0.2	**6.9**		
		13DOF	81	798	16	>64	12.3									1.2	49.4	35.8	1.2	**12.3**		
		36DOF	17	80	32	>64	17.6										35.3	47.1		**17.6**		
	Tiamulin	1DOF	451	799	16	32	N/I					0.2		0.2	1.3	10.4	59.2	28.2	0.4			
		13DOF	81	798	16	32	N/I								3.7	6.2	50.6	29.6	9.9			
		36DOF	17	80	32	> 32	N/I								5.9	5.9	35.3	41.2	11.8			
	Tildipirosin	1DOF	451	799	≤1	≤1	0						91.8	7.8	0.4							
		13DOF	81	798	≤1	2	1.2						82.7	9.9	6.2			**1.2**				
		36DOF	17	80	≤1	2	0						82.4	17.7								
	Tilmicosin	1DOF	451	799	4	8	N/I							16.2	66.3	16.2	1.3					
		13DOF	81	798	4	8	N/I							22.2	53.1	18.5	3.7	**2.5**				
		36DOF	17	80	4	16	N/I							17.7	52.9	17.7	5.9	**5.9**				
	Trimethoprim-sul-famethoxazole	1DOF	451	799	≤2	≤2	N/I							99.3	0.7							
		13DOF	81	798	≤2	≤2	N/I							98.8	1.2							
		36DOF	17	80	≤2	≤2	N/I							94.1	5.9							
	Tulathromycin	1DOF	451	799	≤8	≤8	0									99.8		0.2				
		13DOF	81	798	≤8	≤8	1.2									97.5		1.2		**1.2**		
		36DOF	17	80	≤8	≤8	0									94.1		5.9				
	Tylosin	1DOF	451	799	32	32	N/I					0.2			0.2	1.1	18.9	72.5	7.1			
		13DOF	81	798	32	>32	N/I								1.2	3.7	16.1	66.7	12.4			
		36DOF	17	80	32	>32	N/I										17.7	52.9	29.4			
**III**	Florfenicol	1DOF	451	799	0.5	0.5	0				39.7	60.3										

								Distribution (%) of MICs (µg/mL)														
Category[a]	Antimicrobial	Sampling time	No. Isolates	No. Calves	MIC 50	MIC 90	% R	0.03	0.06	0.12	0.25	0.5	1	2	4	8	16	32	64	128	256	512
		13DOF	81	798	0.5	0.5	0				37.0	63.0										
		36DOF	17	80	0.5	0.5	0				35.3	64.7										
	Sulphadimethoxine	1DOF	451	799	>256	>256	N/I														25.5	74.5
		13DOF	81	798	>256	>256	N/I														35.8	64.2
		36DOF	17	80	>256	>256	N/I														23.5	76.5
	Tetracycline	1DOF	451	799	≤0.5	1	6.2					73.4	17.7	1.8	0.9	**6.0**	**0.2**					
		13DOF	81	798	≤0.5	2	9.8					69.1	18.5	2.5		**8.6**	**1.2**					
		36DOF	17	80	≤0.5	8	17.7					64.7	17.7			**11.8**	**5.9**					

Pathogen MIC breakpoints were based on references available by the Clinical and Laboratory Standards Institute (CLSI) [CLSI VET01S (2023)].

Based on CLSI breakpoints, susceptible isolates are indicated by a white background, intermediate by a medium grey background, and resistant by a dark grey background.

The thick vertical black line indicates the CLSI established resistant breakpoint MIC when available.

The black-outlined cells represent the MIC dilution ranges that were tested. If growth was observed at the highest antimicrobial tested, the MIC was assigned to the next dilution and will appear outside the outlined area, while those with MIC concentrations one dilution lower than the tested range will be situated below it.

[a] Categorization of antimicrobial based on importance in human medicine as defined by the Veterinary Drug Directorate.

[b] For ampicillin, the current breakpoint for susceptible isolates is ≤0.03, 0.06-0.12 for intermediate, and ≥0.25 µg/ml for resistant. However, the lowest concentration available on the sensitivity panel is ≤0.25.

Table S3. Distribution of minimum inhibitory concentrations among *H.somni* isolates from year 2020 tulathromycin-treated calves.

Category[a]	Antimicrobial	Sampling time	No. Isolates	No. Calves	MIC 50	MIC 90	% R	Distribution (%) of MICs (μg/mL)														
								0.03	0.06	0.12	0.25	0.5	1	2	4	8	16	32	64	128	256	512
I	Ceftiofur	1DOF	77	799	≤0.25	≤0.25	0				100											
		13DOF	35	798	≤0.25	≤0.25	0				97.1	2.9										
		36DOF	39	80	≤0.25	≤0.25	0				100											
	Danofloxacin	1DOF	77	799	≤0.12	≤0.12	N/I			97.4	1.3			1.3								
		13DOF	35	798	≤0.12	≤0.12	N/I			100												
		36DOF	39	80	≤0.12	≤0.12	N/I			100												
	Enrofloxacin	1DOF	77	799	≤0.12	≤0.12	0			100												
		13DOF	35	798	≤0.12	≤0.12	0			100												
		36DOF	39	80	≤0.12	≤0.12	0			100												
II	Ampicillin[b]	1DOF	77	799	≤0.25	≤0.25	0				100											
		13DOF	35	798	≤0.25	≤0.25	0				100											
		36DOF	39	80	≤0.25	≤0.25	0				100											
	Clindamycin	1DOF	77	799	1	2	N/I				1.3	20.8	50.7	27.3								
		13DOF	35	798	1	2	N/I				2.9	8.6	65.7	20.0		2.9						
		36DOF	39	80	1	2	N/I				2.6	2.6	69.2	25.6								
	Gamithromycin	1DOF	77	799	≤1	2	0						66.2	33.8								
		13DOF	35	798	≤1	2	2.9						77.1	20.0			**2.9**					
		36DOF	39	80	≤1	2	0						87.2	12.8								
	Gentamicin	1DOF	77	799	16	> 16	N/I								5.2	23.4	46.8	24.7				
		13DOF	35	798	16	> 16	N/I							2.9		37.1	37.1	22.9				
		36DOF	39	80	16	> 16	N/I								2.6	15.4	43.6	38.5				
	Neomycin	1DOF	77	799	32	> 32	N/I										2.6	72.7	24.7			
		13DOF	35	798	32	> 32	N/I								2.9			65.7	31.4			
		36DOF	39	80	> 32	> 32	N/I										2.6	25.6	71.8			

Category[a]	Antimicrobial	Sampling time	No. Isolates	No. Calves	MIC 50	MIC 90	% R	Distribution (%) of MICs (µg/mL)														
								0.03	0.06	0.12	0.25	0.5	1	2	4	8	16	32	64	128	256	512
	Penicillin	1DOF	77	799	≤0.12	≤0.12	0			100												
		13DOF	35	798	≤0.12	≤0.12	0			94.3	2.9	2.9										
		36DOF	39	80	≤0.12	≤0.12	0			100												
	Spectinomycin	1DOF	77	799	32	32	1.3									1.3	20.8	72.7	3.9	**1.3**		
		13DOF	35	798	32	32	0										28.6	71.4				
		36DOF	39	80	32	32	0										20.5	74.4	5.1			
	Tiamulin	1DOF	77	799	2	4	N/I					2.6	13.0	42.9	41.6							
		13DOF	35	798	2	4	N/I						8.6	60.0	28.6		2.9					
		36DOF	39	80	2	4	N/I						20.5	56.4	23.1							
	Tildipirosin	1DOF	77	799	8	16	5.2							6.5	28.6	36.4	**23.4**	**5.2**				
		13DOF	35	798	8	16	5.7						2.9	8.6	17.1	60.0	**5.7**	**5.7**				
		36DOF	39	80	4	16	0							7.7	51.3	28.2	**12.8**					
	Tilmicosin	1DOF	77	799	8	16	N/I							1	12	38	44	5				
		13DOF	35	798	8	16	N/I							3	3	49	46					
		36DOF	39	80	8	16	N/I								13	56	28	3				
	Trimethoprim-sulfamethoxazole	1DOF	77	799	≤2	≤2	N/I							90.9	9.1							
		13DOF	35	798	≤2	≤2	N/I							100								
		36DOF	39	80	≤2	≤2	N/I							97.4	2.6							
	Tulathromycin	1DOF	77	799	32	32	7.8									27.3	19.5	45.5	**7.8**			
		13DOF	35	798	16	32	5.7									20.0	42.9	31.4	**5.7**			
		36DOF	39	80	16	32	0									48.7	18.0	33.3				
	Tylosin	1DOF	77	799	8	16	N/I							6.5	31.2	49.4	13.0					
		13DOF	35	798	8	8	N/I							5.7	28.5	62.9			2.9			
		36DOF	39	80	4	8	N/I							2.6	51.3	46.2						
**III**	Florfenicol	1DOF	77	799	≤0.25	≤0.25	0				98.7	1.3										

Category[a]	Antimicrobial	Sampling time	No. Isolates	No. Calves	MIC 50	MIC 90	% R	Distribution (%) of MICs (μg/mL)														
								0.03	0.06	0.12	0.25	0.5	1	2	4	8	16	32	64	128	256	512
		13DOF	35	798	≤0.25	≤0.25	0				97.1	2.9										
		36DOF	39	80	≤0.25	≤0.25	0				100											
	Sulphadimethoxine	1DOF	77	799	>256	>256	N/I														33.8	66.2
		13DOF	35	798	>256	>256	N/I														20.0	80.0
		36DOF	39	80	>256	>256	N/I														38.5	61.5
	Tetracycline	1DOF	77	799	≤0.5	≤0.5	0					100										
		13DOF	35	798	≤0.5	≤0.5	0					100										
		36DOF	39	80	≤0.5	≤0.5	0					100										

Pathogen MIC breakpoints were based on references available by the Clinical and Laboratory Standards Institute (CLSI) [CLSI VET01S (2023)].

Based on CLSI breakpoints, susceptible isolates are indicated by a white background, intermediate by a medium grey background, and resistant by a dark grey background.

The thick vertical black line indicates the CLSI established resistant breakpoint MIC when available.

The black-outlined cells represent the MIC dilution ranges that were tested. If growth was observed at the highest antimicrobial tested, the MIC was assigned to the next dilution and will appear outside the outlined area, while those with MIC concentrations one dilution lower than the tested range will be situated below it.

[a] Categorization of antimicrobial based on importance in human medicine as defined by the Veterinary Drug Directorate.

[b] For ampicillin, the current breakpoint for susceptible isolates is ≤0.03, 0.06-0.12 for intermediate, and ≥0.25 μg/ml for resistant. However, the lowest concentration available on the sensitivity panel is ≤0.25.

Table S4. Distribution of minimum inhibitory concentrations among *M. haemolytics* isolates from year 2021 tulathromycin-treated calves.

Category[a]	Antimicrobial	Sampling time	No. Isolates	No. Calves	MIC 50	MIC 90	% R	Distribution (%) of MICs (µg/mL)														
								0.03	0.06	0.12	0.25	0.5	1	2	4	8	16	32	64	128	256	512
I	Ceftiofur	1DOF	196	399	≤0.25	≤0.25	0				97.4	1.0	1.0	0.5								
		13DOF	119	399	≤0.25	≤0.25	0				100											
		36DOF	66	119	≤0.25	≤0.25	0				100											
	Danofloxacin	1DOF	196	399	≤0.12	≤0.12	0.5			98.0	0.5	0.5	**1.0**									
		13DOF	119	399	≤0.12	≤0.12	0			100												
		36DOF	66	119	≤0.12	≤0.12	0			100												
	Enrofloxacin	1DOF	196	399	≤ 0.12	≤ 0.12	0			98.0		0.5	1.5									
		13DOF	119	399	≤ 0.12	≤ 0.12	0			100												
		36DOF	66	119	≤ 0.12	≤ 0.12	0			100												
II	Ampicillin[b]	1DOF	196	399	≤0.25	≤0.25	1.0				99.0	**1.0**										
		13DOF	119	399	≤0.25	≤0.25	0				100											
		36DOF	66	119	≤0.25	≤0.25	1.5				98.5	**1.5**										
	Clindamycin	1DOF	196	399	16	16	N/I								3.1	45.4	45.9	5.6				
		13DOF	119	399	16	16	N/I									16.8	81.5	1.7				
		36DOF	66	119	16	16	N/I									12.1	84.8	3.0				
	Gamithromycin	1DOF	196	399	≤1	2	0						60.2	39.3	0.5							
		13DOF	119	399	2	>8	22.7						45.4	16.0	16.0		**22.7**					
		36DOF	66	119	≤1	>8	15.2							54.5	30.3		**15.2**					
	Gentamicin	1DOF	196	399	2	2	N/I						23.5	72.4	3.1	1.0						
		13DOF	119	399	2	2	N/I						9.2	88.2	2.5							
		36DOF	66	119	2	2	N/I						9.1	87.9	1.5	1.5						
	Neomycin	1DOF	196	399	4	4	N/I								91.8	5.1	1.0	0.5	1.5			
		13DOF	119	399	4	>32	N/I								54.6	5.0	0.8	39.5				

Category[a]	Antimicrobial	Sampling time	No. Isolates	No. Calves	MIC 50	MIC 90	% R	Distribution (%) of MICs (µg/mL)														
								0.03	0.06	0.12	0.25	0.5	1	2	4	8	16	32	64	128	256	512
		36DOF	66	119	8	>32	N/I								39.4	19.7		1.5	39.4			
	Penicillin	1DOF	196	399	≤0.12	0.25	0			70.9	25.5	3.6										
		13DOF	119	399	≤0.12	0.25	0			73.1	21.8	5.0										
		36DOF	66	119	≤0.12	0.5	0			53.0	36.4	10.6										
	Spectinomycin	1DOF	196	399	32	32	0											100				
		13DOF	119	399	32	32	0										0.8	99.2				
		36DOF	66	119	32	32	0									1.5	4.5	93.9				
	Tiamulin	1DOF	196	399	16	32	N/I								0.5	5.1	83.2	10.7	0.5			
		13DOF	119	399	16	32	N/I									4.2	81.5	13.4	0.8			
		36DOF	66	119	16	32	N/I									10.6	75.8	13.6				
	Tildipirosin	1DOF	196	399	≤1	2	0.5						71.4	26.5	1.0	0.5	**0.5**					
		13DOF	119	399	≤1	>16	31.9						50.4	9.2		8.4	**10.9**	**21.0**				
		36DOF	66	119	≤1	>16	18.2						53.0	7.6	15.2	6.1	**6.1**	**12.1**				
	Tilmicosin	1DOF	196	399	8	8	1.0							0.5	25.0	69.9	3.6	**1.0**				
		13DOF	119	399	8	>16	36.1								26.1	31.9	5.9	**36.1**				
		36DOF	66	119	8	>16	18.2								45.5	13.6	22.7	**18.2**				
	Trimethoprim-sulfamethoxazole	1DOF	196	399	≤ 2	≤ 2	N/I							100								
		13DOF	119	399	≤ 2	≤ 2	N/I							98.3	1.7							
		36DOF	66	119	≤ 2	≤ 2	N/I							100.0								
	Tulathromycin	1DOF	196	399	≤8	≤ 8	0									98.0	1.0	1.0				
		13DOF	119	399	≤8	>64	22.7									59.7		17.6	**0.8**	**21.8**		
		36DOF	66	119	≤8	>64	15.2									60.6	24.2		**1.5**	**13.6**		
	Tylosin	1DOF	196	399	64	64	N/I										0.5	2.6	96.9			
		13DOF	119	399	64	64	N/I												100			
		36DOF	66	119	64	64	N/I											3.0	97.0			

Category[a]	Antimicrobial	Sampling time	No. Isolates	No. Calves	MIC 50	MIC 90	% R	Distribution (%) of MICs (μg/mL)														
								0.03	0.06	0.12	0.25	0.5	1	2	4	8	16	32	64	128	256	512
III	Florfenicol	1DOF	196	399	1	1	0				1.5	8.2	88.8	1.0	0.5							
		13DOF	119	399	1	1	0				0.8	7.6	90.8	0.8								
		36DOF	66	119	1	1	0				3.0	18.2	78.8									
	Sulphadimethoxine	1DOF	196	399	≤256	>256	N/I														78.1	21.9
		13DOF	119	399	>256	>256	N/I														48.7	51.3
		36DOF	66	119	≤256	>256	N/I														57.6	42.4
	Tetracycline	1DOF	196	399	0.5	0.5	0.5						91.3	7.1	1.0	**0.5**						
		13DOF	119	399	1	8	15.1					38.7	21.0		25.2	**14.3**	**0.8**					
		36DOF	66	119	1	8	34.8					7.6	53.0		4.5	**34.8**						

Pathogen MIC breakpoints were based on references available by the Clinical and Laboratory Standards Institute (CLSI) [CLSI VET01S (2023)].

Based on CLSI breakpoints, susceptible isolates are indicated by a white background, intermediate by a medium grey background, and resistant by a dark grey background.

The thick vertical black line indicates the CLSI established resistant breakpoint MIC when available.

The black-outlined cells represent the MIC dilution ranges that were tested. If growth was observed at the highest antimicrobial tested, the MIC was assigned to the next dilution and will appear outside the outlined area, while those with MIC concentrations one dilution lower than the tested range will be situated below it.

[a] Categorization of antimicrobial based on importance in human medicine as defined by the Veterinary Drug Directorate.

[b] For ampicillin, the current breakpoint for susceptible isolates is ≤0.03, 0.06-0.12 for intermediate, and ≥0.25 μg/ml for resistant. However, the lowest concentration available on the sensitivity panel is ≤0.25.

Table S5. Distribution of minimum inhibitory concentrations among *P. multocida* isolates from year 2021 tulathromycin-treated calves.

								Distribution (%) of MICs (µg/mL)														
**Category[a]**	**Antimicrobial**	**Sampling time**	**No. Isolates**	**No. Calves**	**MIC 50**	**MIC 90**	**% R**	**0.03**	**0.06**	**0.12**	**0.25**	**0.5**	**1**	**2**	**4**	**8**	**16**	**32**	**64**	**128**	**256**	**512**
I	Ceftiofur	1DOF	172	399	≤0.25	≤0.25	0				99.4	1										
		13DOF	47	399	≤0.25	≤0.25	0				100											
		36DOF	25	119	≤0.25	≤0.25	0				96.0		4.0									
	Danofloxacin	1DOF	172	399	≤0.12	≤0.12	0			100												
		13DOF	47	399	≤0.12	≤0.12	0			100												
		36DOF	25	119	≤0.12	≤0.12	0			100												
	Enrofloxacin	1DOF	172	399	≤0.12	≤0.12	0			100												
		13DOF	47	399	≤0.12	≤0.12	0			100												
		36DOF	25	119	≤0.12	≤0.12	0			100												
II	Ampicillin[b]	1DOF	172	399	≤0.25	≤0.25	5.2				94.8	**5.2**										
		13DOF	47	399	≤0.25	≤0.25	12.8				87.2	**12.8**										
		36DOF	25	119	≤0.25	0.5	0.0				100											
	Clindamycin	1DOF	172	399	>16	>16	N/I							0.6		1.7	5.8	91.9				
		13DOF	47	399	>16	>16	N/I										6.4	93.6				
		36DOF	25	119	>16	>16	N/I						4.0				24.0	72.0				
	Gamithromycin	1DOF	172	399	1	1	0						90.7	8.7	0.6							
		13DOF	47	399	1	1	0						97.9		2.1							
		36DOF	25	119	1	1	0						92.0		4.0	4.0						
	Gentamicin	1DOF	172	399	4	8	N/I						0.6	19.2	36.6	43.6						
		13DOF	47	399	4	8	N/I							23.4	36.2	40.4						
		36DOF	25	119	4	8	N/I						12.0	12.0	68.0	8.0						
	Neomycin	1DOF	172	399	16	32	N/I								21.5	15.1	50.0	10.5	2.9			
		13DOF	47	399	16	32	N/I								27.7	14.9	36.2	17.0	4.3			
		36DOF	25	119	16	32	N/I									24.0	16.0	56.0	4.0			

Category[a]	Antimicrobial	Sampling time	No. Isolates	No. Calves	MIC 50	MIC 90	% R	Distribution (%) of MICs (μg/mL)														
								0.03	0.06	0.12	0.25	0.5	1	2	4	8	16	32	64	128	256	512
	Penicillin	1DOF	172	399	≤0.12	0.25	0			75.0	23.8	1.2										
		13DOF	47	399	≤0.12	0.25	0			72.3	27.7											
		36DOF	25	119	≤0.12	0.25	0			84.0	12.0	4.0										
	Spectinomycin	1DOF	172	399	32	32	2.9									0.6	2.3	92.4	1.7	**2.9**		
		13DOF	47	399	32	32	0									2.1	21.3	74.5	2.1			
		36DOF	25	119	32	32	0									12.0	84.0	4.0				
	Tiamulin	1DOF	172	399	32	32	N/I					0.6	0.6		1.7	5.2	41.3	44.2	6.4			
		13DOF	47	399	32	>32	N/I								2.1	8.5	29.8	48.9	10.6			
		36DOF	25	119	32	>32	N/I							4.0		16.0	52.0	28.0				
	Tildipirosin	1DOF	172	399	2	2	0						50.0	44.8	5.2							
		13DOF	47	399	≤1	2	0						59.6	31.9	6.4	2.1						
		36DOF	25	119	≤1	2	0						88.0	12.0								
	Tilmicosin	1DOF	172	399	8	8	N/I							4.7	30.2	59.9	5.2					
		13DOF	47	399	4	8	N/I							4.3	48.9	38.3	6.4	2.1				
		36DOF	25	119	4	8	N/I							24.0	56.0	12.0	4.0	4.0				
	Trimethoprim-sulfamethoxazole	1DOF	172	399	2	2	N/I							99.4	0.6							
		13DOF	47	399	2	2	N/I							97.9	2.1							
		36DOF	25	119	2	2	N/I							100								
	Tulathromycin	1DOF	172	399	≤8	≤8	0									99.4	0.6					
		13DOF	47	399	≤8	≤8	0									97.9	2.1					
		36DOF	25	119	≤8	≤8	0									96.0	4.0					
	Tylosin	1DOF	172	399	32	>32	N/I							0.6	0.6	1.7	2.3	52.3	42.4			
		13DOF	47	399	32	>32	N/I									2.1	10.6	55.3	31.9			
		36DOF	25	119	32	>32	N/I								4.0	4.0	32.0	44.0	16.0			
**III**	Florfenicol	1DOF	172	399	0.5	0.5	0				16.3	78.5	5.2									

Category[a]	Antimicrobial	Sampling time	No. Isolates	No. Calves	MIC 50	MIC 90	% R	0.03	0.06	0.12	0.25	0.5	1	2	4	8	16	32	64	128	256	512
								Distribution (%) of MICs (µg/mL)														
		13DOF	47	399	0.5	0.5	0				29.8	70.2										
		36DOF	25	119	0.5	0.5	0				40.0	60.0										
	Sulphadimethoxine	1DOF	172	399	>256	>256	N/I														16.9	83.1
		13DOF	47	399	>256	>256	N/I														25.5	74.5
		36DOF	25	119	>256	>256	N/I														60.0	40.0
	Tetracycline	1DOF	172	399	≤0.5	1	1.7					87.2	8.1	2.3	0.6	**1.7**						
		13DOF	47	399	≤0.5	≤0.5	0					95.7		4.3								
		36DOF	25	119	≤0.5	≤0.5	0					100										

Pathogen MIC breakpoints were based on references available by the Clinical and Laboratory Standards Institute (CLSI) [CLSI VET01S (2023)].

Based on CLSI breakpoints, susceptible isolates are indicated by a white background, intermediate by a medium grey background, and resistant by a dark grey background.

The thick vertical black line indicates the CLSI established resistant breakpoint MIC when available.

The black-outlined cells represent the MIC dilution ranges that were tested. If growth was observed at the highest antimicrobial tested, the MIC was assigned to the next dilution and will appear outside the outlined area, while those with MIC concentrations one dilution lower than the tested range will be situated below it.

[a] Categorization of antimicrobial based on importance in human medicine as defined by the Veterinary Drug Directorate.

[b] For ampicillin, the current breakpoint for susceptible isolates is ≤0.03, 0.06-0.12 for intermediate, and ≥0.25 µg/ml for resistant. However, the lowest concentration available on the sensitivity panel is ≤0.25.

Table S6. Distribution of minimum inhibitory concentrations among *H. somni* isolates from year 2021 tulathromycin-treated calves.

Category[a]	Antimicrobial	Sampling time	No. Isolates	No. Calves	MIC 50	MIC 90	% R	Distribution (%) of MICs (μg/mL)														
								0.03	0.06	0.12	0.25	0.5	1	2	4	8	16	32	64	128	256	512
I	Ceftiofur	1DOF	37	399	≤0.25	≤0.25	0				91.9	8.1										
		13DOF	40	399	≤0.25	≤0.25	0				97.5	2.5										
		36DOF	69	119	≤0.25	≤0.25	0				100											
	Danofloxacin	1DOF	37	399	≤0.12	≤0.12	0			100												
		13DOF	40	399	≤0.12	≤0.12	0			97.5	2.5											
		36DOF	69	119	≤0.12	≤0.12	0			100												
	Enrofloxacin	1DOF	37	399	≤0.12	≤0.12	0			100												
		13DOF	40	399	≤0.12	≤0.12	0			100												
		36DOF	69	119	≤0.12	≤0.12	0			100												
II	Ampicillin[b]	1DOF	37	399	≤0.25	≤0.25	0				100											
		13DOF	40	399	≤0.25	≤0.25	2.5				97.5		**2.5**									
		36DOF	69	119	≤0.25	≤0.25	4.3				95.7	**4.3**										
	Clindamycin	1DOF	37	399	1	1	N/I				2.7	27.0	62.2	8.1								
		13DOF	40	399	1	2	N/I					17.5	42.5	40.0								
		36DOF	69	119	1	2	N/I				1.4	29.0	59.4	10.1								
	Gamithromycin	1DOF	37	399	≤1	≤1	0						100									
		13DOF	40	399	≤1	2	0						90.0	10.0								
		36DOF	69	119	≤1	≤1	0						98.6		1.4							
	Gentamicin	1DOF	37	399	16	>16	N/I								5.4	27.0	45.9	21.6				
		13DOF	40	399	16	16	N/I								5.0	17.5	70.0	7.5				
		36DOF	69	119	16	>16	N/I										33.3	66.7				
	Neomycin	1DOF	37	399	>32	>32	N/I										2.7	32.4	64.9			
		13DOF	40	399	>32	>32	N/I										2.5	20.0	77.5			
		36DOF	69	119	>32	>32	N/I											33.3	66.7			

Category[a]	Antimicrobial	Sampling time	No. Isolates	No. Calves	MIC 50	MIC 90	% R	Distribution (%) of MICs (μg/mL)														
								0.03	0.06	0.12	0.25	0.5	1	2	4	8	16	32	64	128	256	512
	Penicillin	1DOF	37	399	≤0.12	≤0.12	0			97.3	2.7											
		13DOF	40	399	≤0.12	≤0.12	0			100												
		36DOF	69	119	≤0.12	≤0.12	1.4			91.3	2.9	4.3	**1.4**									
	Spectinomycin	1DOF	37	399	32	32	2.7									5.4	29.7	59.5	2.7	**2.7**		
		13DOF	40	399	32	32	2.5									2.5	32.5	60.0	2.5	**2.5**		
		36DOF	69	119	16	32	0									5.8	49.3	43.5	1.4			
	Tiamulin	1DOF	37	399	2	2	N/I					5.4	43.2	45.9	5.4							
		13DOF	40	399	2	4	N/I						27.5	40.0	32.5							
		36DOF	69	119	2	2	N/I						44.9	47.8	7.2							
	Tildipirosin	1DOF	37	399	2	8	0						5.4	48.6	18.9	27.0						
		13DOF	40	399	8	8	0						5.0	17.5	15.0	62.5						
		36DOF	69	119	2	8	0						17.4	47.8	13.0	21.7						
	Tilmicosin	1DOF	37	399	4	8	N/I							16	38	41	5					
		13DOF	40	399	8	16	N/I							10	15	43	33					
		36DOF	69	119	4	8	N/I							14	48	29	9					
	Trimethoprim-sulfamethoxazole	1DOF	37	399	2	2	N/I							97.3	2.7							
		13DOF	40	399	2	2	N/I							100								
		36DOF	69	119	2	2	N/I							100								
	Tulathromycin	1DOF	37	399	8	16	0									89.2	10.8					
		13DOF	40	399	16	16	0									50.0	47.5	2.5				
		36DOF	69	119	8	16	0									68.1	29.0	2.9				
	Tylosin	1DOF	37	399	4	8	N/I						5.4	13.5	62.2	18.9						
		13DOF	40	399	8	8	N/I							20.0	25.0	52.5	2.5					
		36DOF	69	119	4	8	N/I							34.8	47.8	15.9	1.4					
**III**	Florfenicol	1DOF	37	399	≤0.25	≤0.25	0				100											

								Distribution (%) of MICs (µg/mL)														
**Category[a]**	**Antimicrobial**	**Sampling time**	**No. Isolates**	**No. Calves**	**MIC 50**	**MIC 90**	**% R**	**0.03**	**0.06**	**0.12**	**0.25**	**0.5**	**1**	**2**	**4**	**8**	**16**	**32**	**64**	**128**	**256**	**512**
		13DOF	40	399	≤0.25	≤0.25	0				100											
		36DOF	69	119	≤0.25	≤0.25	0				94.2	1.4	4.3									
	Sulphadimethoxine	1DOF	37	399	>256	>256	N/I														2.7	97.3
		13DOF	40	399	>256	>256	N/I															100
		36DOF	69	119	>256	>256	N/I														24.6	75.4
	Tetracycline	1DOF	37	399	≤0.5	≤0.5	0					97.3	2.7									
		13DOF	40	399	≤0.5	≤0.5	2.5					97.5					**2.5**					
		36DOF	69	119	≤0.5	1	8.7					89.9	1.4	2.9	5.8							

Pathogen MIC breakpoints were based on references available by the Clinical and Laboratory Standards Institute (CLSI) [CLSI VET01S (2023)].

Based on CLSI breakpoints, susceptible isolates are indicated by a white background, intermediate by a medium grey background, and resistant by a dark grey background.

The thick vertical black line indicates the CLSI established resistant breakpoint MIC when available.

The black-outlined cells represent the MIC dilution ranges that were tested. If growth was observed at the highest antimicrobial tested, the MIC was assigned to the next dilution and will appear outside the outlined area, while those with MIC concentrations one dilution lower than the tested range will be situated below it.

[a] Categorization of antimicrobial based on importance in human medicine as defined by the Veterinary Drug Directorate.

[b] For ampicillin, the current breakpoint for susceptible isolates is ≤0.03, 0.06-0.12 for intermediate, and ≥0.25 µg/ml for resistant. However, the lowest concentration available on the sensitivity panel is ≤0.25.

Table S7. Distribution of minimum inhibitory concentrations among *M. haemolytica* isolates from year 2021 oxytetracycline-treated calves.

								Distribution (%) of MICs (μg/mL)														
**Category[a]**	**Antimicrobial**	**Sampling time**	**No. Isolates**	**No. Calves**	**MIC 50**	**MIC 90**	**% R**	**0.03**	**0.06**	**0.12**	**0.25**	**0.5**	**1**	**2**	**4**	**8**	**16**	**32**	**64**	**128**	**256**	**512**
I	Ceftiofur	1DOF	192	399	≤0.25	≤0.25	0				99.5	0.5										
		13DOF	219	398	≤0.25	≤0.25	0				99.1		0.5	0.5								
		36DOF	32	110	≤0.25	≤0.25	0				100											
	Danofloxacin	1DOF	192	399	≤0.12	≤0.12	0			100												
		13DOF	219	398	≤0.12	≤0.12	0			99.5	0.5											
		36DOF	32	110	≤0.12	≤0.12	0			100.0												
	Enrofloxacin	1DOF	192	399	≤0.12	≤0.12	0			99.0	1.0											
		13DOF	219	398	≤0.12	≤0.12	0			100												
		36DOF	32	110	≤0.12	≤0.12	0			100												
II	Ampicillin[b]	1DOF	192	399	≤0.25	≤0.25	0				100											
		13DOF	219	398	≤0.25	≤0.25	0.5				99.5		**0.5**									
		36DOF	32	110	≤0.25	≤0.25	0				100											
	Clindamycin	1DOF	192	399	8	16	N/I									51.0	47.4	1.6				
		13DOF	219	398	16	16	N/I							0.5		22.4	73.5	3.7				
		36DOF	32	110	16	16	N/I								3.1	40.6	53.1	3.1				
	Gamithromycin	1DOF	192	399	≤1	2	0						62.5	37.5								
		13DOF	219	398	≤1	2	1.4						80.4	18.3			**1.4**					
		36DOF	32	110	≤1	2	0						9.4	90.6								
	Gentamicin	1DOF	192	399	2	2	N/I						29.2	69.8	1.0							
		13DOF	219	398	2	2	N/I						16.4	81.7	1.8							
		36DOF	32	110	2	2	N/I						9.4	90.6								
	Neomycin	1DOF	192	399	≤4	≤4	N/I								97.4	2.6						
		13DOF	219	398	≤4	≤4	N/I								91.3	7.3			1.4			

								Distribution (%) of MICs (μg/mL)														
**Category[a]**	**Antimicrobial**	**Sampling time**	**No. Isolates**	**No. Calves**	**MIC 50**	**MIC 90**	**% R**	**0.03**	**0.06**	**0.12**	**0.25**	**0.5**	**1**	**2**	**4**	**8**	**16**	**32**	**64**	**128**	**256**	**512**
		36DOF	32	110	≤4	≤4	N/I								90.6	9.4						
	Penicillin	1DOF	192	399	≤0.12	0.5	0.5			60.4	28.1	10.9	**0.5**									
		13DOF	219	398	≤0.12	0.5	0.9			56.2	32.0	11.0	**0.5**	**0.5**								
		36DOF	32	110	0.25	0.5	0			46.9	40.6	12.5										
	Spectinomycin	1DOF	192	399	32	32	0.5										0.5	99.0		**0.5**		
		13DOF	219	398	32	32	0										0.9	98.6	0.5			
		36DOF	32	110	32	32	0											100				
	Tiamulin	1DOF	192	399	16	32	N/I									8.3	79.7	10.9	1.0			
		13DOF	219	398	16	32	N/I									11.0	75.8	12.8	0.5			
		36DOF	32	110	16	16	N/I									12.5	84.4	3.1				
	Tildipirosin	1DOF	192	399	≤1	2	0						70.8	28.1	1.0							
		13DOF	219	398	≤1	2	1.4						87.2	10.5	0.9			**1.4**				
		36DOF	32	110	≤1	2	0						71.9	28.1								
	Tilmicosin	1DOF	192	399	8	8	0								22.4	70.8	6.8					
		13DOF	219	398	8	8	1.4							0.5	35.6	58.0	4.6	**1.4**				
		36DOF	32	110	8	8	0								46.9	46.9	6.3					
	Trimethoprim-sulfamethoxazole	1DOF	192	399	2	2	N/I							99.0	1.0							
		13DOF	219	398	2	2	N/I							100								
		36DOF	32	110	2	2	N/I							100								
	Tulathromycin	1DOF	192	399	≤8	≤8	0									100						
		13DOF	219	398	≤8	≤8	1.4									98.6				**1.4**		
		36DOF	32	110	≤8	≤8	0									100						
	Tylosin	1DOF	192	399	>32	>32	N/I												0.5	99.5		
		13DOF	219	398	>32	>32	N/I					0.5								99.5		
		36DOF	32	110	>32	>32	N/I												100.0			

Category[a]	Antimicrobial	Sampling time	No. Isolates	No. Calves	MIC 50	MIC 90	% R	0.03	0.06	0.12	0.25	0.5	1	2	4	8	16	32	64	128	256	512
								Distribution (%) of MICs (μg/mL)														
III	Florfenicol	1DOF	192	399	1	1	0					7.3	92.2	0.5								
		13DOF	219	398	1	1	0					10.5	89.0	0.5								
		36DOF	32	110	1	1	0				3.1	18.8	75.0	3.1								
	Sulphadimethoxine	1DOF	192	399	≤256	≤256	N/I														94.3	5.7
		13DOF	219	398	≤256	>256	N/I														77.2	22.8
		36DOF	32	110	≤256	>256	N/I														78.1	21.9
	Tetracycline	1DOF	192	399	≤0.5	≤0.5	0					98.4	1.0	0.5								
		13DOF	219	398	≤0.5	1	1.4					71.2	27.4			**1.4**						
		36DOF	32	110	1	1	0					50.0	50.0									

Pathogen MIC breakpoints were based on references available by the Clinical and Laboratory Standards Institute (CLSI) [CLSI VET01S (2023)].

Based on CLSI breakpoints, susceptible isolates are indicated by a white background, intermediate by a medium grey background, and resistant by a dark grey background.

The thick vertical black line indicates the CLSI established resistant breakpoint MIC when available.

The black-outlined cells represent the MIC dilution ranges that were tested. If growth was observed at the highest antimicrobial tested, the MIC was assigned to the next dilution and will appear outside the outlined area, while those with MIC concentrations one dilution lower than the tested range will be situated below it.

[a] Categorization of antimicrobial based on importance in human medicine as defined by the Veterinary Drug Directorate.

[b] For ampicillin, the current breakpoint for susceptible isolates is ≤0.03, 0.06-0.12 for intermediate, and ≥0.25 μg/ml for resistant. However, the lowest concentration available on the sensitivity panel is ≤0.25.

Table S8. Distribution of minimum inhibitory concentrations among *P. multocida* isolates from year 2021 oxytetracycline-treated calves.

Category[a]	Antimicrobial	Sampling time	No. Isolates	No. Calves	MIC 50	MIC 90	% R	Distribution (%) of MICs (μg/mL)														
								0.03	0.06	0.12	0.25	0.5	1	2	4	8	16	32	64	128	256	512
I	Ceftiofur	1DOF	159	399	≤0.25	≤0.25	0				99.4	0.6										
		13DOF	154	398	≤0.25	≤0.25	0				99.4	0.6										
		36DOF	40	110	≤0.25	≤0.25	0				97.5		2.5									
	Danofloxacin	1DOF	159	399	≤0.12	≤0.12	0			100												
		13DOF	154	398	≤0.12	≤0.12	0			100												
		36DOF	40	110	≤0.12	≤0.12	0			100												
	Enrofloxacin	1DOF	159	399	≤0.12	≤0.12	0			100												
		13DOF	154	398	≤0.12	≤0.12	0			100												
		36DOF	40	110	≤0.12	≤0.12	0			100												
II	Ampicillin[b]	1DOF	159	399	≤0.25	≤0.25	5.0				95.0	**5.0**										
		13DOF	154	398	≤0.25	≤0.25	1.9				98.1	**1.9**										
		36DOF	40	110	≤0.25	≤0.25	5.0				95.0	**2.5**	**2.5**									
	Clindamycin	1DOF	159	399	>16	>16	N/I								3.1	4.4	7.5	84.9				
		13DOF	154	398	>16	>16	N/I							1.3	1.9	1.9	5.8	89.0				
		36DOF	40	110	>16	>16	N/I							2.5	2.5	5.0	7.5	82.5				
	Gamithromycin	1DOF	159	399	1	1	0						93.1	6.9								
		13DOF	154	398	1	1	0						93.5	6.5								
		36DOF	40	110	1	1	0						95.0	5.0								
	Gentamicin	1DOF	159	399	4	8	N/I						1.3	12.6	73.0	13.2						
		13DOF	154	398	4	8	N/I						1.3	9.7	76.6	12.3						
		36DOF	40	110	4	8	N/I						5.0	5.0	55.0	32.5	2.5					
	Neomycin	1DOF	159	399	16	16	N/I								8.2	34.0	49.1	4.4	4.4			

Category[a]	Antimicrobial	Sampling time	No. Isolates	No. Calves	MIC 50	MIC 90	% R	Distribution (%) of MICs (µg/mL)														
								0.03	0.06	0.12	0.25	0.5	1	2	4	8	16	32	64	128	256	512
		13DOF	154	398	16	>32	N/I								7.1	39.0	40.9	1.3	11.7			
		36DOF	40	110	16	>32	N/I								7.5	17.5	52.5	7.5	15.0			
	Penicillin	1DOF	159	399	≤0.12	0.25	0			87.4	12.6											
		13DOF	154	398	≤0.12	0.25	0			89.6	10.4											
		36DOF	40	110	≤0.12	0.25	2.5			90.0	7.5		**2.5**									
	Spectinomycin	1DOF	159	399	32	32	6.9									1.9	27.7	62.3	1.3	**6.9**		
		13DOF	154	398	32	>64	34.4									0.6	28.6	36.4		**34.4**		
		36DOF	40	110	32	>64	37.5									5.0	22.5	32.5	2.5	**37.5**		
	Tiamulin	1DOF	159	399	16	32	N/I					0.6	0.6	3.1	2.5	13.8	45.9	29.6	3.8			
		13DOF	154	398	16	32	N/I					0.6	0.6	1.3	1.3	13.0	34.4	46.8	1.9			
		36DOF	40	110	32	32	N/I					2.5	2.5	2.5	2.5	10.0	22.5	55.0	2.5			
	Tildipirosin	1DOF	159	399	≤1	2	0						65.4	34.0	0.6							
		13DOF	154	398	≤1	2	0						74.7	24.7	0.6							
		36DOF	40	110	≤1	2	0						37.5	62.5								
	Tilmicosin	1DOF	159	399	4	8	N/I							11.9	45.9	34.6	7.5					
		13DOF	154	398	4	8	N/I							7.8	44.2	43.5	4.5					
		36DOF	40	110	8	16	N/I							12.5	20.0	57.5	10.0					
	Trimethoprim-sulfamethoxazole	1DOF	159	399	2	2	N/I							100								
		13DOF	154	398	2	2	N/I							100								
		36DOF	40	110	2	2	N/I							97.5	2.5							
	Tulathromycin	1DOF	159	399	≤8	≤8	0									100						
		13DOF	154	398	≤8	≤8	0									100						
		36DOF	40	110	≤8	≤8	0									100						
	Tylosin	1DOF	159	399	32	>32	N/I							2.5	2.5	2.5	12.6	53.5	26.4			

Category[a]	Antimicrobial	Sampling time	No. Isolates	No. Calves	MIC 50	MIC 90	% R	Distribution (%) of MICs (μg/mL)														
								**0.03**	**0.06**	**0.12**	**0.25**	**0.5**	**1**	**2**	**4**	**8**	**16**	**32**	**64**	**128**	**256**	**512**
		13DOF	154	398	32	>32	N/I							1.3	1.3	1.3	11.0	42.9	42.2			
		36DOF	40	110	>32	>32	N/I								10.0		7.5	30.0	52.5			
III	Florfenicol	1DOF	159	399	0.5	0.5	0				27.7	70.4	1.9									
		13DOF	154	398	0.5	0.5	0				25.3	74.0	0.6									
		36DOF	40	110	0.5	0.5	0				2.5	90.0	7.5									
	Sulphadimethoxine	1DOF	159	399	>256	>256	N/I														40.3	59.7
		13DOF	154	398	>256	>256	N/I														25.3	74.7
		36DOF	40	110	>256	>256	N/I														25.0	75.0
	Tetracycline	1DOF	159	399	≤0.5	2	5.7					79.9	9.4	3.8	1.3	**5.7**						
		13DOF	154	398	≤0.5	8	33.8					54.5	9.1	1.9	0.6	**33.8**						
		36DOF	40	110	1	8	42.5					47.5	10.0			**40.0**	**2.5**					

Pathogen MIC breakpoints were based on references available by the Clinical and Laboratory Standards Institute (CLSI) [CLSI VET01S (2023)].

Based on CLSI breakpoints, susceptible isolates are indicated by a white background, intermediate by a medium grey background, and resistant by a dark grey background.

The thick vertical black line indicates the CLSI established resistant breakpoint MIC when available.

The black-outlined cells represent the MIC dilution ranges that were tested. If growth was observed at the highest antimicrobial tested, the MIC was assigned to the next dilution and will appear outside the outlined area, while those with MIC concentrations one dilution lower than the tested range will be situated below it.

[a] Categorization of antimicrobial based on importance in human medicine as defined by the Veterinary Drug Directorate.

[b] For ampicillin, the current breakpoint for susceptible isolates is ≤0.03, 0.06-0.12 for intermediate, and ≥0.25 μg/ml for resistant. However, the lowest concentration available on the sensitivity panel is ≤0.25.

Table S9. Distribution of minimum inhibitory concentrations among *H. somni* isolates from year 2021 oxytetracycline-treated calves.

Category[a]	Antimicrobial	Sampling time	No. Isolates	No. Calves	MIC 50	MIC 90	% R	Distribution (%) of MICs (μg/mL)														
								0.03	0.06	0.12	0.25	0.5	1	2	4	8	16	32	64	128	256	512
I	Ceftiofur	1DOF	17	399	≤0.25	≤0.25	0				100											
		13DOF	46	398	≤0.25	≤0.25	0				95.7	2.2	2.2									
		36DOF	76	110	≤0.25	≤0.25	0				100											
	Danofloxacin	1DOF	17	399	≤0.12	≤0.12	N/I			100												
		13DOF	46	398	≤0.12	≤0.12	N/I			97.8			2.2									
		36DOF	76	110	≤0.12	≤0.12	N/I			98.7	1.3											
	Enrofloxacin	1DOF	17	399	≤0.12	≤0.12	0			100												
		13DOF	46	398	≤0.12	≤0.12	0			95.7	2.2		2.2									
		36DOF	76	110	≤0.12	≤0.12	0			98.7	1.3											
II	Ampicillin[b]	1DOF	17	399	≤0.25	≤0.25	0				100											
		13DOF	46	398	≤0.25	≤0.25	0				100											
		36DOF	76	110	≤0.25	≤0.25	1.3				98.7	**1.3**										
	Clindamycin	1DOF	17	399	1	1	N/I					17.6	82.4									
		13DOF	46	398	1	1	N/I					8.7	89.1	2.2								
		36DOF	76	110	1	1	N/I					25.0	73.7	1.3								
	Gamithromycin	1DOF	17	399	≤1	≤1	0						100									
		13DOF	46	398	≤1	≤1	0						100									
		36DOF	76	110	≤1	≤1	0						100									
	Gentamicin	1DOF	17	399	16	>16	N/I									35.3	47.1	17.6				
		13DOF	46	398	>16	>16	N/I									4.3	34.8	60.9				
		36DOF	76	110	16	>16	N/I								1.3	7.9	44.7	46.1				
	Neomycin	1DOF	17	399	>32	>32	N/I											23.5	76.5			
		13DOF	46	398	>32	>32	N/I										2.2	4.3	93.5			

Category[a]	Antimicrobial	Sampling time	No. Isolates	No. Calves	MIC 50	MIC 90	% R	0.03	0.06	0.12	0.25	0.5	1	2	4	8	16	32	64	128	256	512
								**Distribution (%) of MICs (µg/mL)**														
		36DOF	76	110	>32	>32	N/I											10.5	89.5			
	Penicillin	1DOF	17	399	≤0.12	≤0.12	0			100												
		13DOF	46	398	≤0.12	≤0.12	0			97.8		2.2										
		36DOF	76	110	≤0.12	≤0.12	0			97.4	1.3	1.3										
	Spectinomycin	1DOF	17	399	32	32	0									5.9	23.5	70.6				
		13DOF	46	398	32	32	0									2.2	8.7	89.1				
		36DOF	76	110	32	32	0									1.3	26.3	71.1	1.3			
	Tiamulin	1DOF	17	399	1	2	N/I					11.8	64.7	23.5								
		13DOF	46	398	1	2	N/I					6.5	54.3	30.4	8.7							
		36DOF	76	110	1	2	N/I						64.5	35.5								
	Tildipirosin	1DOF	17	399	2	8	0						23.5	41.2	23.5	11.8						
		13DOF	46	398	2	8	0						4.3	65.2	10.9	17.4	**2.2**					
		36DOF	76	110	2	8	0						5.3	50.0	34.2	10.5						
	Tilmicosin	1DOF	17	399	4	8	N/I							18	35	41	6					
		13DOF	46	398	4	8	N/I							11	59	26	4					
		36DOF	76	110	4	8	N/I							9	47	38	3	3				
	Trimethoprim-sulfamethoxazole	1DOF	17	399	2	2	N/I							100								
		13DOF	46	398	2	2	N/I							100								
		36DOF	76	110	2	2	N/I							97.4	2.6							
	Tulathromycin	1DOF	17	399	8	32	0									64.7	23.5	11.8				
		13DOF	46	398	8	16	0									76.1	23.9					
		36DOF	76	110	8	16	0									75.0	22.4	2.6				
	Tylosin	1DOF	17	399	4	4	N/I						5.9	41.2	47.1	5.9						
		13DOF	46	398	4	8	N/I							30.4	56.5	13.0						

Category[a]	Antimicrobial	Sampling time	No. Isolates	No. Calves	MIC 50	MIC 90	% R	Distribution (%) of MICs (μg/mL)														
								**0.03**	**0.06**	**0.12**	**0.25**	**0.5**	**1**	**2**	**4**	**8**	**16**	**32**	**64**	**128**	**256**	**512**
		36DOF	76	110	4	8	N/I							25.0	56.6	18.4						
III	Florfenicol	1DOF	17	399	≤0.25	≤0.25	0				100											
		13DOF	46	398	≤0.25	≤0.25	0				95.7	4.3										
		36DOF	76	110	≤0.25	≤0.25	0				92.1	7.9										
	Sulphadimethoxine	1DOF	17	399	>256	>256	N/I														23.5	76.5
		13DOF	46	398	>256	>256	N/I														4.3	95.7
		36DOF	76	110	>256	>256	N/I														15.8	84.2
	Tetracycline	1DOF	17	399	≤0.5	≤0.5	5.9					94.1				**5.9**						
		13DOF	46	398	≤0.5	>8	30.4					69.6				**2.2**	**28.3**					
		36DOF	76	110	≤0.5	>8	14.5					85.5				**2.6**	**11.8**					

Pathogen MIC breakpoints were based on references available by the Clinical and Laboratory Standards Institute (CLSI) [CLSI VET01S (2023)].

Based on CLSI breakpoints, susceptible isolates are indicated by a white background, intermediate by a medium grey background, and resistant by a dark grey background.

The thick vertical black line indicates the CLSI established resistant breakpoint MIC when available.

The black-outlined cells represent the MIC dilution ranges that were tested. If growth was observed at the highest antimicrobial tested, the MIC was assigned to the next dilution and will appear outside the outlined area, while those with MIC concentrations one dilution lower than the tested range will be situated below it.

[a] Categorization of antimicrobial based on importance in human medicine as defined by the Veterinary Drug Directorate.

[b] For ampicillin, the current breakpoint for susceptible isolates is ≤0.03, 0.06-0.12 for intermediate, and ≥0.25 μg/ml for resistant. However, the lowest concentration available on the sensitivity panel is ≤0.25.

Supplementary Materials S2- Simulation Model to Evaluate Sample Size Estimates in Support of Pen-Level Testing

From:

Variation in Pen-Level Prevalence of BRD Bacterial Pathogens and Antimicrobial Resistance Following Feedlot Arrival in Beef Calves

Jennifer N. Abi Younes [1], John R. Campbell [1], Simon J. G. Otto [2], Sheryl P. Gow [3], Amelia R. Woolums [4], Murray Jelinski [1], Stacey Lacoste [1] and Cheryl L. Waldner [1,*]

[1] Department of Large Animal Clinical Sciences, Western College of Veterinary Medicine, University of Saskatchewan, Saskatoon, SK S7N 5B4, Canada; jennifer.abiyounes@usask.ca (J.N.A.Y.); john.campbell@usask.ca (J.R.C.); murray.jelinski@usask.ca (M.J.); srl676@mail.usask.ca (S.L.)
[2] School of Public Health, University of Alberta, Edmonton, AB T6G 1C9, Canada; simon.otto@ualberta.ca
[3] Canadian Integrated Program for Antimicrobial Resistance Surveillance, Public Health Agency of Canada, Saskatoon, SK S7L 0Z2, Canada; sheryl.gow@phac-aspc.ca
[4] Department of Pathobiology and Population Medicine, College of Veterinary Medicine, Mississippi State University, Mississippi State, MS 39762, USA; amelia.woolums@msstate.edu
* Correspondence: cheryl.waldner@usask.ca

Objective

The objective of this supplementary study was to investigate the potential for a sample of calves from a pen to describe the true proportion of calves in that pen with AMR of interest.

Methods

Association between sample size per pen and expected prevalence information

Recognizing that whole pen sampling as presented in this study is not practical for routine use in a commercial feedlot environment, an exploratory simulation-based approach was used to investigate proposed sample sizes per pen, the resulting apparent prevalence of calves with the antimicrobial-resistant pathogens of interest from each sample size, and the range of uncertainty in the associated true prevalence for the calves in the pen. A commercial software tool (AnyLogic® 8, The AnyLogic Company, www.anylogic.com) was used to build a simulation model of calves within a feedlot pen, with options that made it possible to vary the number of calves in the pen, number of calves randomly sampled from the pen, and the true prevalence of the target pathogen with AMR of interest in the pen (i.e., the number of calves with the antimicrobial-resistant pathogen of interest divided by the total number of calves). From the model simulations, the number of true positive calves that were detected within the pen-level sample was recorded for each iteration of various sampling scenarios. The simulation assumed the clinical sensitivity and specificity of the test were 100% in the current exploratory analysis.

Based on discussions with private feedlot veterinarians and veterinarians participating in the CIPARS feedlot AMR program, sample sizes of 10, 20, and 30 animals per pen were deemed a potential reasonable range for consideration, given the expected time required to collect samples. Surveillance conducted by CIPARS has historically sampled 16 calves per pen (47), but 20 calves were sampled per pen in the fall of 2022 (Sheryl Gow, personal communication).

In this exploratory study, we conducted simulations using a working pen size of 200 calves. Simulations were completed with 10,000 iterations per run. We explored various pen-level prevalence values, treating prevalence as a single parameter (i.e., number of calves with resistant bacteria/ total of calves). The range of pen-level prevalence values

included increments of 0.5% within the 0.5 to 5% range (representing 1 positive case out of 200 calves, 2 out of 200, 10 out of 200, and so on) and then in 5% intervals from 5 to 100% (20 out of 200, 30 out of 200, 40 out of 200, and so forth, up to 200 out of 200).

The model generated the counts of true positives identified in each pen sample for each iteration. The resulting data were summarized to determine the predicted probabilities (i.e., the sample apparent prevalence) across all iterations of detecting each possible number of true positives per sample size for each subsequent scenario of total true positives in the pen. The resulting 90% prediction intervals (5th to 95th percentiles) for the apparent prevalence were summarized, graphed, and compared for each sample size and for each pen true prevalence scenario to demonstrate the expected uncertainty in prediction of true pen prevalence based on the sample.

Results

Simulation of pen-level sampling

The results of the simulation exercise suggest that, even at a sample size of 30 calves per pen of 200, a random sample had limited power to provide precise estimates of true pen prevalence for the simulated outcome (Figure S1), based on the widths of the prediction intervals in population true prevalence compared to the sample apparent prevalence. Notable improvement was achieved in the precision of the estimates of true prevalence when the sample size per pen increased from 10 to 20. However, while a sample size of 30 did result in some improvement of precision compared to 20, the improvement was substantially less than the increase from 10 to 20 per pen.

If zero samples are positive from a sample of 10 calves per pen of 200, there is a 95% chance the true pen-level prevalence is <30% (Figure S1). If zero samples are positive from a sample of 20 calves per pen of 200, the corresponding value is <15% prevalence, and for 30 calves sampled per pen of 200 calves the result is <10%. As a result, even with a sample size of 30 calves, it is not possible to discriminate between any pen-level prevalence of <10%.

To follow a specific case example based on the findings of this simulation experiment where 20 samples per pen of 200 calves were used for surveillance, sufficient information was provided to differentiate relatively low pen-level prevalence from moderate pen-level prevalence from high pen-level prevalence (Figure S1). A finding of 0 positives out of 20 samples resulted in a 90% predictive interval for true pen prevalence of 0 to 15%. Seven positives out of 20 resulted in a 90% predictive interval for true pen prevalence of between 20 and 55%. Finally, a finding of 16 positives out of 20 samples resulted in a 90% predictive interval for true pen prevalence of between 60 and 95%.

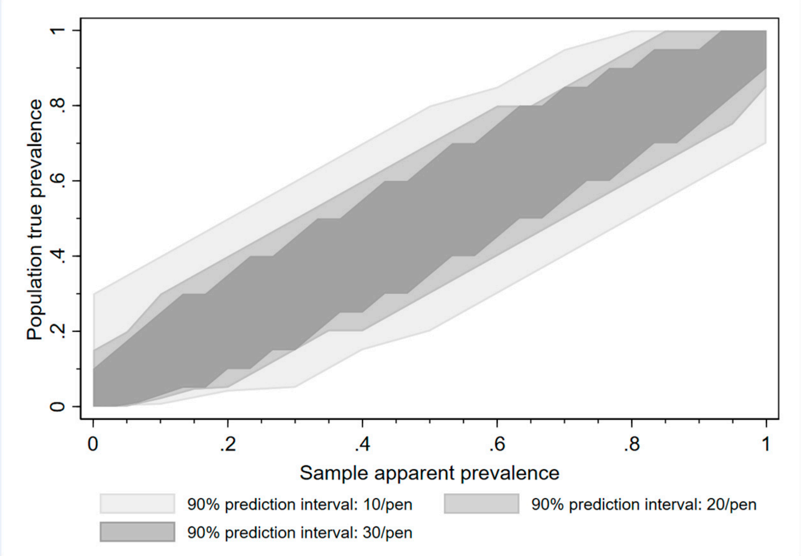

Figure S1. Exploratory simulation of the association between apparent sample-level prevalence and 90% prediction intervals for true pen-level prevalence for the detection of a calves with an antimicrobial-resistant pathogen using different intensities of pen-level sampling. Pen size = 200 calves, with test sensitivity and specificity assumed to be 100%.

Discussion

In exploring the number of calf samples necessary to estimate the pen-level prevalence of variables of interest, traditional sample size formulas were unable to provide meaningful results. The hierarchical nature of calves being aggregated within pens violated the assumed independence of samples required in these formulas. Two approaches for generating sample size calculations for clustered data in animal subjects have been described: 1) inflation of a crude sample size estimate using a design effect, and 2) the use of a simulation-based model [52,53]. The robust data generated by this study allowed for real-world results to be compared to the results of the simulation model. The limitations in the practical number of animals sampled resulted in the lack of precision surrounding pen-level prevalence estimates from the simulation model in this exploratory analysis. However, the model was able to provide a general impression of the pen-level risks of using the results of a sample size of 20 calves per pen by differentiating into low, medium, and high risk. The need for these distinctions was supported by the wide range in pen-level prevalence estimates reported in the current study, particularly at 13DOF near the time of highest treatment risk for calves having received metaphylaxis. This information remains valuable as such laboratory-based data can provide evidence towards individual pen management and antimicrobial needs.

The simulation model used for this exploratory investigation on pen-level sample sizes assumed perfect (100%) sensitivity and specificity for culture results for simplicity and because the true clinical values for BRD bacteria detection are currently unknown. However, future research on culture sensitivity and specificity for detecting bacteria of interest would allow for adjustment of the sensitivity/specificity values. This research group intends to address the issue of unknown sensitivity/specificity of culture by applying a Bayesian model approach to evaluate culture diagnostics in the absence of a gold standard using the real-world culture results of BRD bacteria generated by the current study. Future simulation models on sample sizes could then incorporate these true sensitivity and specificity values for improved predictions.
